# Supplementary material for: Single-Cell Profiling Defines Transcriptomic Signatures Specific to Tumor-Reactive versus Virus-Responsive CD4+ T Cells
Source: Cell Rep. Author manuscript; Available in PMC 2019 Dec 27. (PMC6934378; doi:10.1016/j.celrep.2019.10.131)
Supplement: 1 [file NIHMS1545825-supplement-1.pdf]

Cell Reports, Volume *29*

Supplemental Information

Single-Cell Profiling Defines Transcriptomic Signatures Specific to Tumor-Reactive versus Virus-Responsive $CD4^+$ T Cells

Assaf Magen, Jia Nie, Thomas Ciucci, Samira Tamoutounour, Yongmei Zhao, Monika Mehta, Bao Tran, Dorian B. McGavern, Sridhar Hannenhalli, and Rémy Bosselut

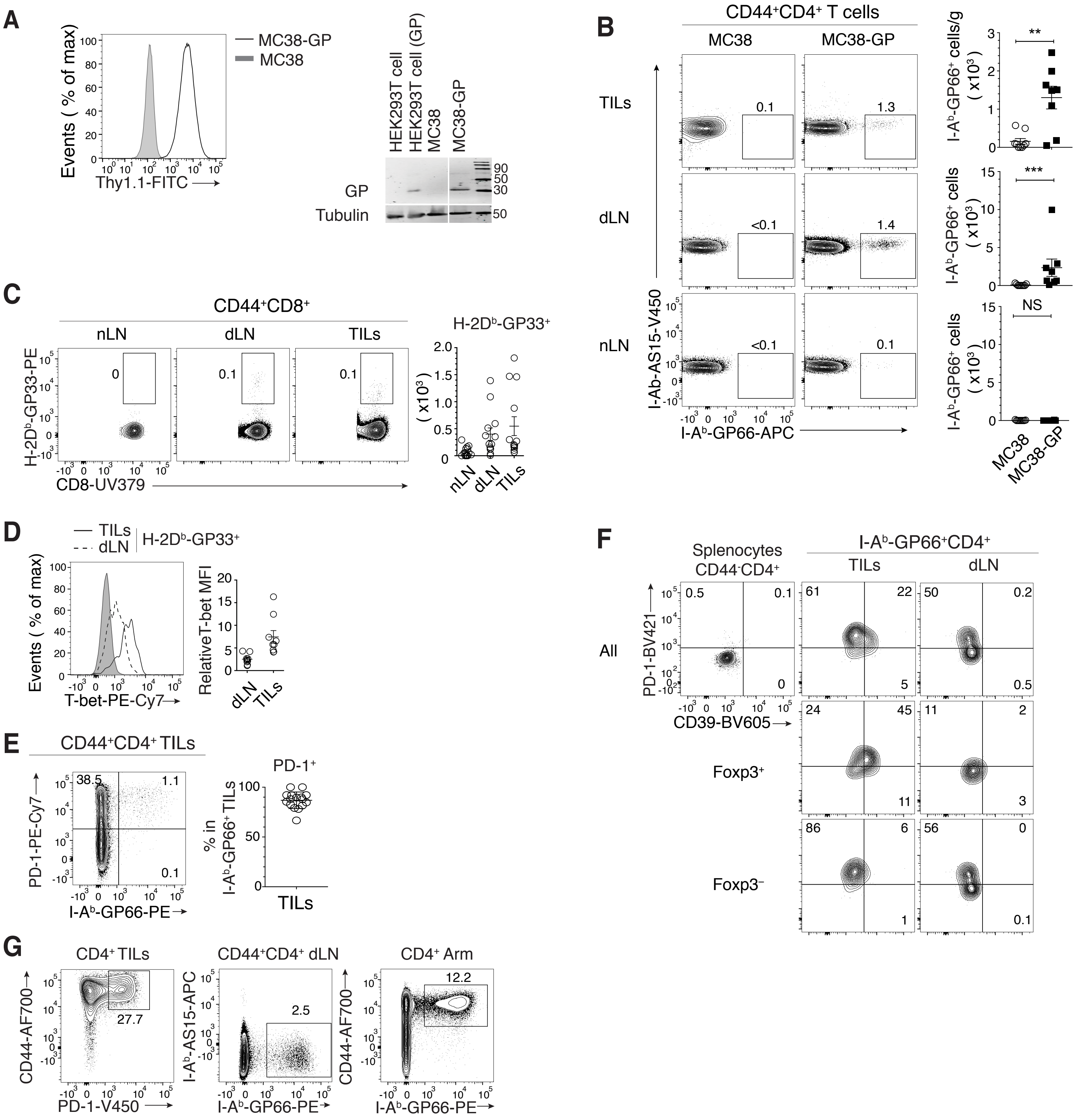
A
Events (% of max)
Thy1.1-FITC
MC38-GP
MC38
HEK293T cell
HEK293T cell (GP)
MC38
MC38-GP
GP
Tubulin
90
50
30
50
B
CD44+CD4+ T cells
MC38
MC38-GP
TILs
dLN
nLN
0.1
1.3
<0.1
1.4
<0.1
0.1
I-Ab-AS15-V450
I-Ab-GP66-APC
I-Ab-GP66+ cells/g (x10^3)
I-Ab-GP66+ cells (x10^3)
**
***
NS
MC38
MC38-GP
C
CD44+CD8+
nLN
dLN
TILs
0
0.1
0.1
H-2Db-GP33-PE
CD8-UV379
H-2Db-GP33+
(x10^3)
nLN
dLN
TILs
D
TILs
dLN
H-2Db-GP33+
Events (% of max)
T-bet-PE-Cy7
Relative T-bet MFI
dLN
TILs
E
CD44+CD4+ TILs
38.5
1.1
0.1
PD-1-PE-Cy7
I-Ab-GP66-PE
PD-1+
% in I-Ab-GP66+ TILs
TILs
F
Splenocytes CD44-CD4+
I-Ab-GP66+CD4+
TILs
dLN
All
Foxp3+
Foxp3–
PD-1-BV421
CD39-BV605
0.5
0.1
0
61
22
5
50
0.2
0.5
24
45
11
11
2
3
86
6
1
56
0
0.1
G
CD4+ TILs
CD44+CD4+ dLN
CD4+ Arm
27.7
2.5
12.2
CD44-AF700
PD-1-V450
I-Ab-AS15-APC
I-Ab-GP66-PE

Figure S1. Characterization of antigen-specific CD4$^+$ T cell responses in MC38 colon adenocarcinoma tumors, Related to Figure 1.

(**A**) Overlaid protein expression of Thy1.1 in MC38 and MC38-GP cells (left). Immunoblot analysis of GP protein expression in HEK293T cells, HEK293T cells transfected with pMRX-GP-IRES-Thy1.1 plasmid, MC38 cells or MC38-GP cells (right).

(B) C57BL/6 mice were subcutaneously injected MC38 or MC38-GP cells and analyzed at day 14 post-injection. Left panel shows flow cytometry contour plots of GP66 vs. control (AS15 peptide from *T. gondii*) class II tetramer staining in TILs, dLN and nLN from MC38 and MC38-GP tumor-bearing mice. Right panel shows the number of GP66$^+$ TILs per gram of tumor and total number of GP66$^+$ dLN and nLN cells, separately for MC38 and MC38-GP tumor-bearing mice (Unpaired Mann-Whitney U test, ** $p < 0.01$, *** $p < 0.001$, NS: not significant). Data is from 8 mice of each condition analyzed in two experiments.

(C) Flow cytometry analysis of CD8 expression vs. GP33 class I tetramer staining in nLN ,dLN and TILs from MC38-GP tumor-bearing mice (left). Total number of GP33$^+$ dLN and nLN cells, and number of GP33$^+$ TILs per gram of tumor (right). Data is from 13 mice analyzed in three experiments.

(D) Overlaid protein expression of Tbet in GP33$^+$ CD8$^+$ dLN and TILs; grey shaded histogram shows T-bet expression in naive CD8$^+$ splenocytes from tumor-free control mice; histogram shows concatenated data from 6 individual mice analyzed in one experiment. Graph on the right shows T-bet MFI in GP33$^+$ dLN and TILs; data is from 9 tumor bearing mice analyzed in two experiment and is expressed relative to naive CD8$^+$ splenocytes from tumor-free control mice. Each symbol represents an individual mouse.

(E) Flow cytometry contour plots of GP66 tetramer vs. PD-1 staining in TILs (left) and percentage of PD-1$^+$ cells out of GP66$^+$ TILs (right). Data is from 16 mice analyzed in three experiments.

(F) Right two columns show flow cytometry expression of PD-1 vs. CD39 in GP66$^+$CD4$^+$ TILs or dLN, gated on all such cells (top row) or Foxp3$^+$or Foxp3$^-$ subsets (bottom rows). Top left plot shows staining of naïve splenocytes from tumor-free mice. Data is representative from 11 mice analyzed in three experiments.

(G) GP66-specific CD44^{hi} CD4$^+$ splenocytes were isolated from WT animals 7 days post-infection with LCMV Armstrong. Protein expression contour of populations used for scRNAseq captures from MC38-GP tumor-bearing mice (TILs PD-1 vs. CD44, dLN GP66 vs. AS15 control) and LCMV Armstrong infected mice (GP66 vs. CD44). Data is representative from >50 mice analyzed in >10 experiments.

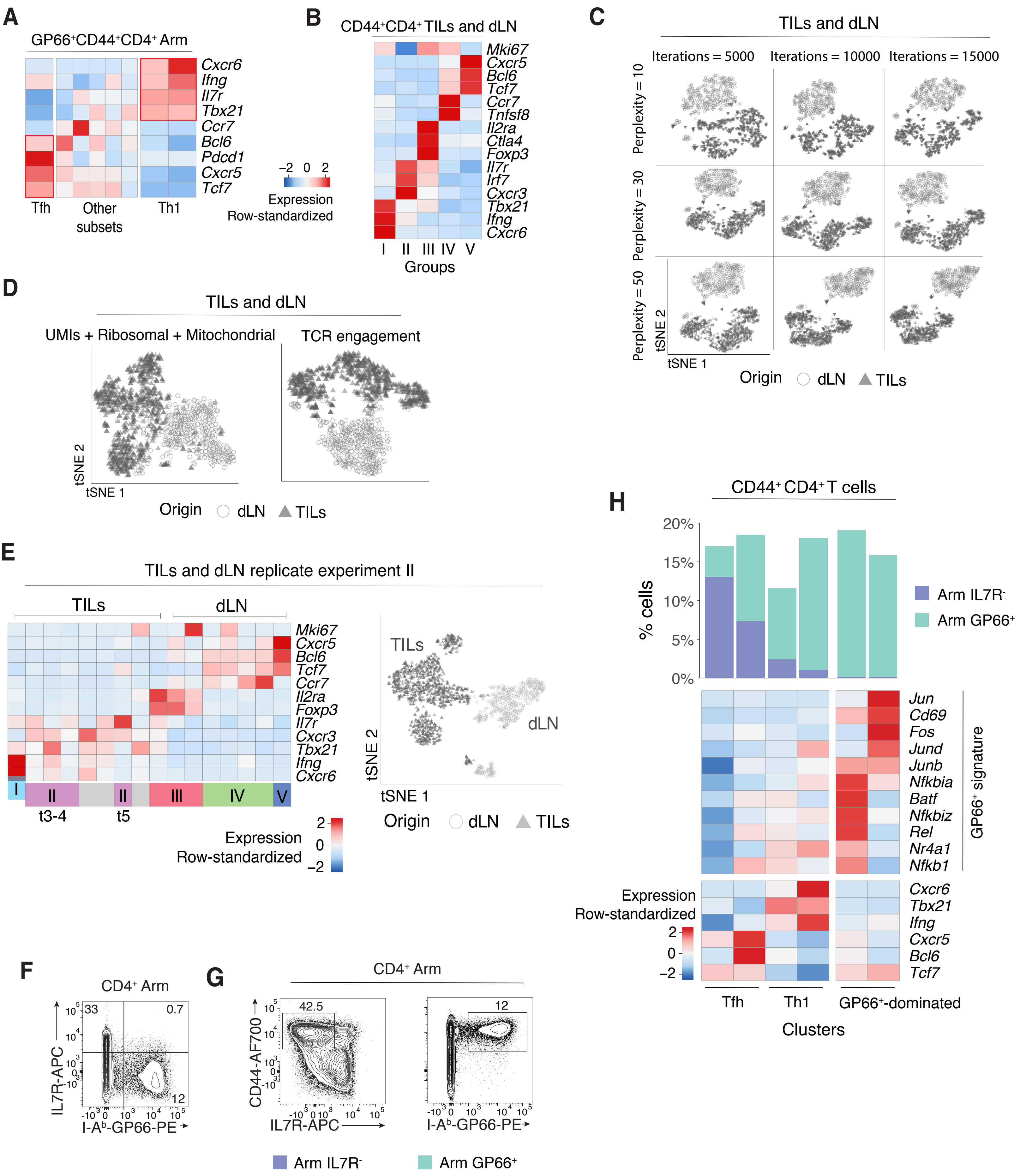
A
GP66+CD44+CD4+ Arm
Cxcr6
Ifng
Il7r
Tbx21
Ccr7
Bcl6
Pdcd1
Cxcr5
Tcf7
Tfh
Other subsets
Th1
−2 0 2
Expression
Row-standardized
B
CD44+CD4+ TILs and dLN
Mki67
Cxcr5
Bcl6
Tcf7
Ccr7
Tnfsf8
Il2ra
Ctla4
Foxp3
Il7r
Irf7
Cxcr3
Tbx21
Ifng
Cxcr6
I II III IV V
Groups
C
TILs and dLN
Iterations = 5000
Iterations = 10000
Iterations = 15000
Perplexity = 10
Perplexity = 30
Perplexity = 50
tSNE 2
tSNE 1
Origin
dLN
TILs
D
TILs and dLN
UMIs + Ribosomal + Mitochondrial
TCR engagement
tSNE 2
tSNE 1
Origin
dLN
TILs
E
TILs and dLN replicate experiment II
TILs
dLN
Mki67
Cxcr5
Bcl6
Tcf7
Ccr7
Il2ra
Foxp3
Il7r
Cxcr3
Tbx21
Ifng
Cxcr6
I
II
II
III
IV
V
t3-4
t5
Expression
Row-standardized
2
0
−2
TILs
dLN
tSNE 2
tSNE 1
Origin
dLN
TILs
F
CD4+ Arm
33
0.7
12
IL7R-APC
I-Ab-GP66-PE
G
CD4+ Arm
42.5
12
CD44-AF700
IL7R-APC
I-Ab-GP66-PE
Arm IL7R-
Arm GP66+
H
CD44+ CD4+ T cells
20%
15%
10%
5%
0%
% cells
Arm IL7R-
Arm GP66+
Jun
Cd69
Fos
Jund
Junb
Nfkbia
Batf
Nfkbiz
Rel
Nr4a1
Nfkb1
GP66+ signature
Cxcr6
Tbx21
Ifng
Cxcr5
Bcl6
Tcf7
Expression
Row-standardized
2
0
−2
Tfh
Th1
GP66+-dominated
Clusters

Figure S2. Characterization of immune responses to Arm and MC38-GP by scRNAseq, Related to figures 1 and 2.

(A) GP66-specific $CD4^+$ splenocytes from WT animals 7 days post-infection with LCMV Armstrong analyzed by scRNAseq. Heatmap shows row-standardized expression of selected genes across Arm clusters.

(B-E) TILs and dLN cells from WT mice at day 14 post MC38-GP injection analyzed by scRNAseq. **(B)** Heatmap shows row-standardized expression of selected genes across main TIL and dLN groups (as defined in text). **(C)** tSNE display of TILs and dLN cells generated using different parameter combination of perplexity and number of iterations, grey-shaded by tissue origin. **(D)** tSNE displays of TILs and dLN cells, grey-shaded by tissue origin, post confounder correction for number of unique molecular identifiers (UMIs) and expression of ribosomal and mitochondrial coding genes (left) or TCR engagement on dLN cells as a result of GP66-tetramer-based purification (right). **(E)** scRNAseq analysis of TILs and dLN cells from replicate experiment II. Heatmap shows row-standardized expression of selected genes across TIL and dLN clusters (left). tSNE display of TILs and dLN cells, grey-shaded by tissue origin (right).

(F-H) Analysis of $CD4^+$ splenocytes from C57BL/6 animals 7 days post-infection with LCMV Armstrong (Arm). **(F)** Flow cytometry contour plot of GP66 tetramer staining vs. IL7R in $CD4^+$ Arm cells. **(G)** Flow cytometry contour plots of IL7R vs. CD44 (for Arm $IL7R^-$ sample, **left**) and GP66 vs. CD44 (for Arm $GP66^+$ sample, **right**). **(H)** Arm $IL7R^-$ and Arm $GP66^+$ cells analyzed by scRNAseq. Heatmap shows row-standardized expression of selected genes across pooled Arm $IL7R^-$ and Arm $GP66^+$ clusters (**bottom**). Bar plot indicates the number of Arm $IL7R^-$ and Arm $GP66^+$ cells in each cluster relative to the total number of cells (**top**).

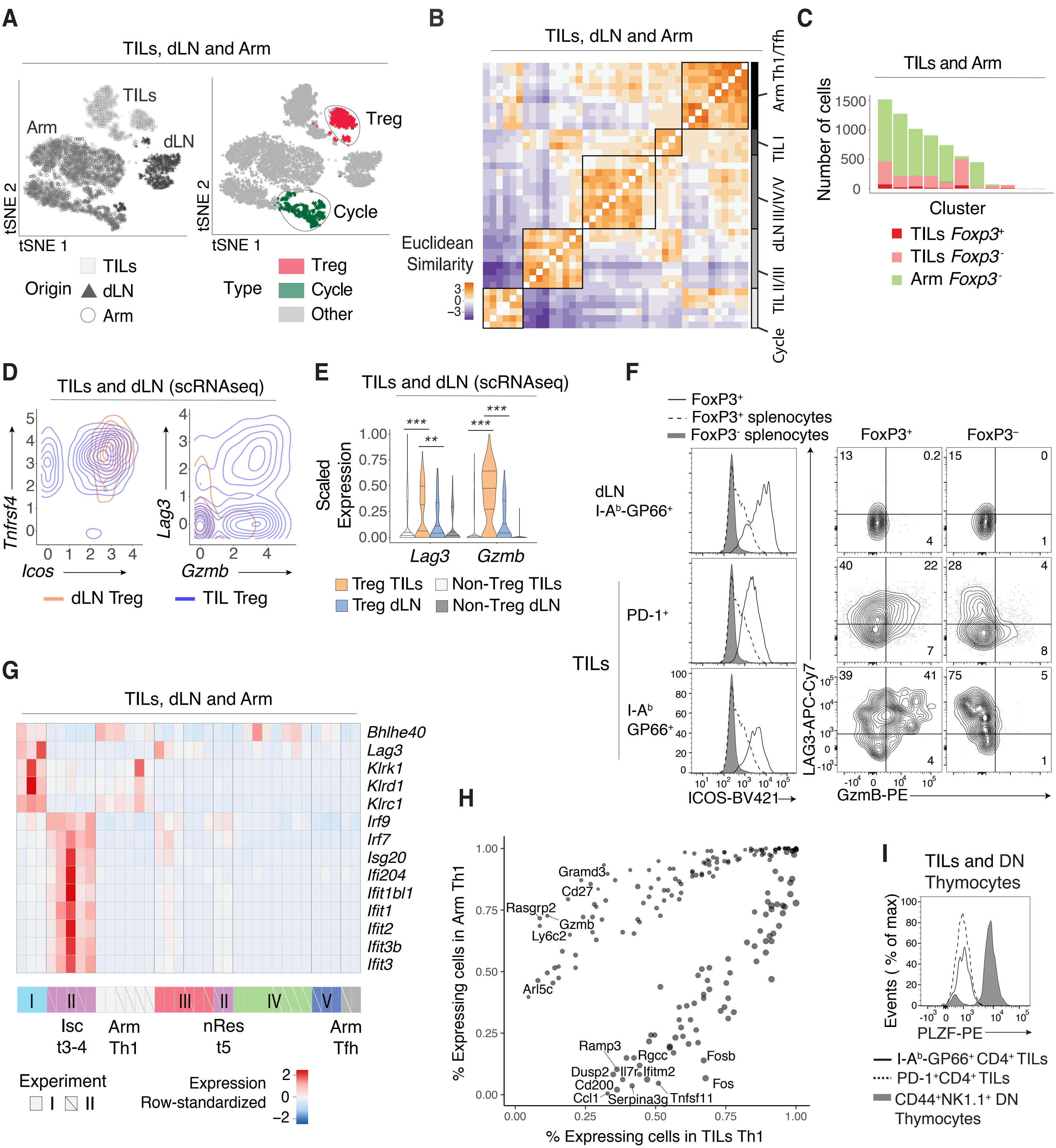
A
TILs, dLN and Arm
TILs
Arm
dLN
tSNE 2
tSNE 1
Treg
Cycle
Origin
TILs
dLN
Arm
Type
Treg
Cycle
Other
B
TILs, dLN and Arm
Euclidean
Similarity
3
0
−3
Arm Th1/Tfh
TIL I
dLN III/IV/V
TIL II/III
Cycle
C
TILs and Arm
Number of cells
1500
1000
500
0
Cluster
TILs Foxp3+
TILs Foxp3-
Arm Foxp3-
D
TILs and dLN (scRNAseq)
Tnfrsf4
Icos
Lag3
Gzmb
dLN Treg
TIL Treg
E
TILs and dLN (scRNAseq)
Scaled
Expression
1.00
0.75
0.50
0.25
0.00
***
**
***
***
Lag3
Gzmb
Treg TILs
Non-Treg TILs
Treg dLN
Non-Treg dLN
F
FoxP3+
FoxP3+ splenocytes
FoxP3- splenocytes
FoxP3+
FoxP3−
dLN
I-Ab-GP66+
TILs
PD-1+
I-Ab
GP66+
13
0.2
4
15
0
1
40
22
7
28
4
8
39
41
4
75
5
1
ICOS-BV421
LAG3-APC-Cy7
GzmB-PE
G
TILs, dLN and Arm
Bhlhe40
Lag3
Klrk1
Klrd1
Klrc1
Irf9
Irf7
Isg20
Ifi204
Ifit1bl1
Ifit1
Ifit2
Ifit3b
Ifit3
I
II
III
II
IV
V
Isc
t3-4
Arm
Th1
nRes
t5
Arm
Tfh
Experiment
I
II
Expression
Row-standardized
2
0
−2
H
% Expressing cells in Arm Th1
% Expressing cells in TILs Th1
Gramd3
Cd27
Rasgrp2
Gzmb
Ly6c2
Arl5c
Ramp3
Rgcc
Fosb
Dusp2
Il7r
Ifitm2
Cd200
Fos
Ccl1
Serpina3g
Tnfsf11
I
TILs and DN
Thymocytes
Events (% of max)
PLZF-PE
I-Ab-GP66+ CD4+ TILs
PD-1+CD4+ TILs
CD44+NK1.1+ DN
Thymocytes

Figure S3. Assessment of tissue-context-specific effects on clustering analyses and TILs-dLN heterogeneity, Related to Figure 3.

(A-C, G,H) TILs, dLN and Arm cells from replicate experiments I and II analyzed by scRNAseq. **(A)** tSNE plots show TILs, dLN, and Arm cells, grey-shaded by origin (left) or color-coded by Treg or cell-cycle (Cycle) clustering assignment (grey for all other clusters) (right). **(B)** Heatmap shows Euclidean similarity between cluster-specific average expression vectors (as defined in text) (left) annotated with cluster origin and cluster group or type (right). **(C)** Bar plot shows relative cluster composition of Foxp3^{+} or Foxp3^{-} TILs and Foxp3^{-} Arm (no Foxp3^{+} cells found in GP66^{+} Arm) after applying a data integration approach (Butler et al., 2018).

(D-E) Comparison of dLN Tregs and TIL Tregs (respectively clusters t6-7 and n1 as shown in Fig. 1A). **(D)** Contour plots of dLN Treg (orange) or TIL Treg (blue) cell distribution according to scRNAseq-detected normalized expression of *Icos* vs. *Tnfrsf4* (left) and *Gzmb* vs. *Lag3* (right). **(E)** Violin plot of *Lag3* and *Gzmb* scRNAseq expression in Treg vs. non-Treg TIL and dLN populations (Unpaired T test, ** $p < 0.01$, *** $p < 0.001$); bands indicate quartiles (25th, 50th and 75th quantile).

(F) Overlaid flow cytometry expression of ICOS in Foxp3^{+} TILs and dLN cells and Foxp3^{+} or Foxp3^{-} CD4^{+} splenocytes from tumor-free control mice (left). Data is representative from 11 mice analyzed in three experiments. Flow cytometry contour plots of Granzyme B vs. LAG3 in Foxp3^{+} TILs or Foxp3^{+} dLN cells (middle), and Foxp3^{-} TILs or Foxp3^{-} dLN (right). Graphs were generated from 5 concatenated files, each of them representing a single mouse and processed in parallel in one experiment. Data is representative of 2 such experiments.

(G) Heatmap shows row-standardized expression of TIL Isc and Th1 characteristic genes across TIL, dLN and Arm clusters.

(H) Gene detection statistics (% expressing cells) of differentially expressed genes by scRNAseq in TILs Th1 and Arm Th1.

(I) Overlaid protein expression of PLZF in GP66^{+} and PD-1^{+} TILs and CD44^{hi} NK1.1^{+} DN (double negative CD4^{-}CD8^{-}) thymocytes from tumor-free control mice. Data is representative from 10 mice analyzed in two experiments.

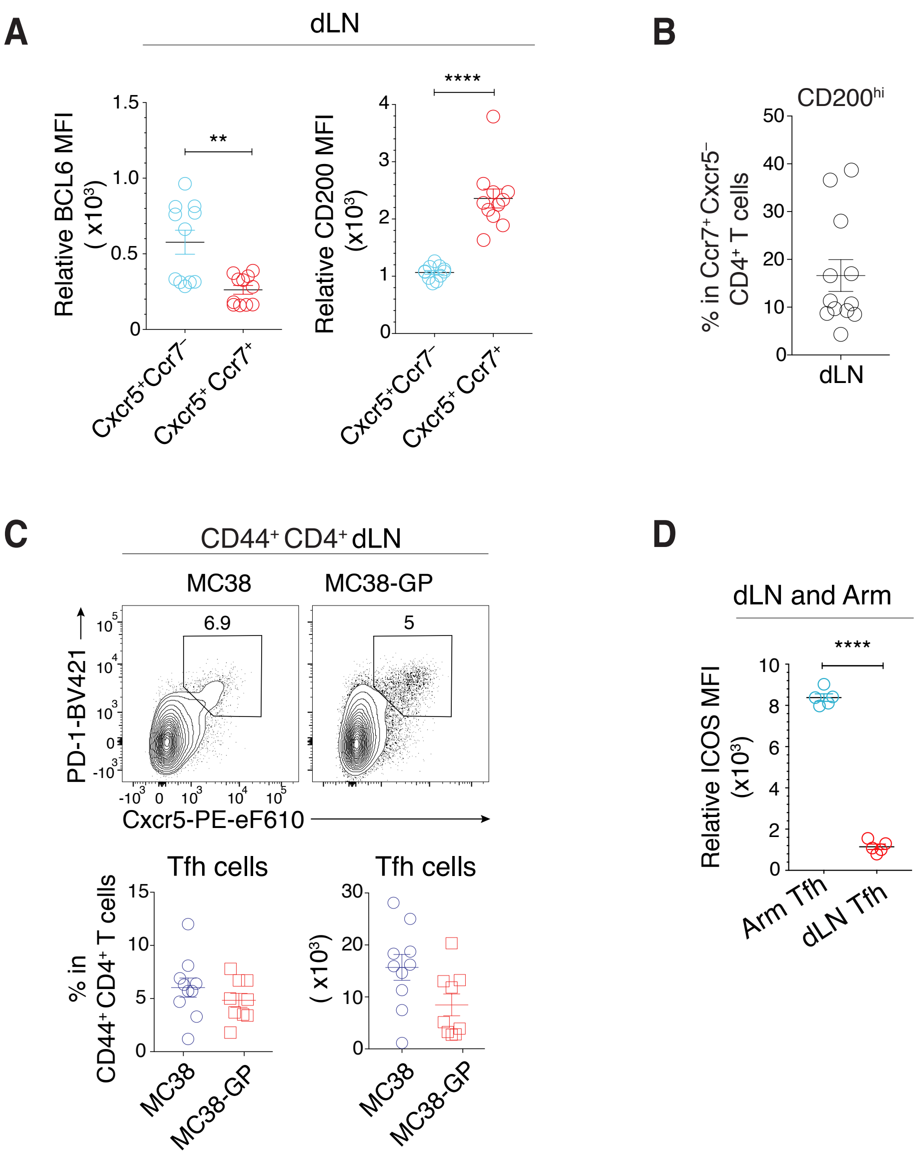
A
dLN
Relative BCL6 MFI
(x10³)
1.5
1.0
0.5
0
**
Cxcr5⁺Ccr7⁻
Cxcr5⁺Ccr7⁺
Relative CD200 MFI
(x10³)
4
3
2
1
0
****
Cxcr5⁺Ccr7⁻
Cxcr5⁺Ccr7⁺
B
CD200hi
% in Ccr7⁺Cxcr5⁻
CD4⁺ T cells
50
40
30
20
10
0
dLN
C
CD44⁺CD4⁺dLN
MC38
MC38-GP
6.9
5
PD-1-BV421
Cxcr5-PE-eF610
Tfh cells
% in
CD44⁺CD4⁺ T cells
15
10
5
0
MC38
MC38-GP
Tfh cells
(x10³)
30
20
10
0
MC38
MC38-GP
D
dLN and Arm
Relative ICOS MFI
(x10³)
****
10
8
6
4
2
0
Arm Tfh
dLN Tfh

Figure S4. dLN cell diversity, Related to Figure 4.

(A) Mean fluorescence intensity (MFI) of BCL6 and CD200 in $CXCR5^+$ or $CCR7^+GP66^+$ dLN cells relative to naive $CD4^+$ splenocytes from tumor-free control mice. (Unpaired t-test, ** $p < 0.005$, **** $p < 0.0001$).

(B) Percentage of $CD200^{hi}$ cells out of $CCR7^+CXCR5^+$ dLN cells.

(C) Top panel shows flow cytometry contour plots of CXCR5 vs. PD-1 in $CD44^{hi}$ $CD4^+$ dLN cells from MC38 and MC38-GP tumor-bearing mice. Bottom panel shows percentage of Tfh cells out of total $CD44^{hi}$ $CD4^+$ T cells in dLN (left) and total number of Tfh cells (right). (A-C) Data is representative of 17 mice analyzed in three experiments.

(D) Mean fluorescence intensity (MFI) levels of ICOS in Arm Tfh and dLN Tfh relative to naive $CD4^+$ splenocytes from tumor-free control mice (Unpaired t-test, $p < 10^{-5}$). (A-C) Data is representative of 10 mice analyzed in two experiments.

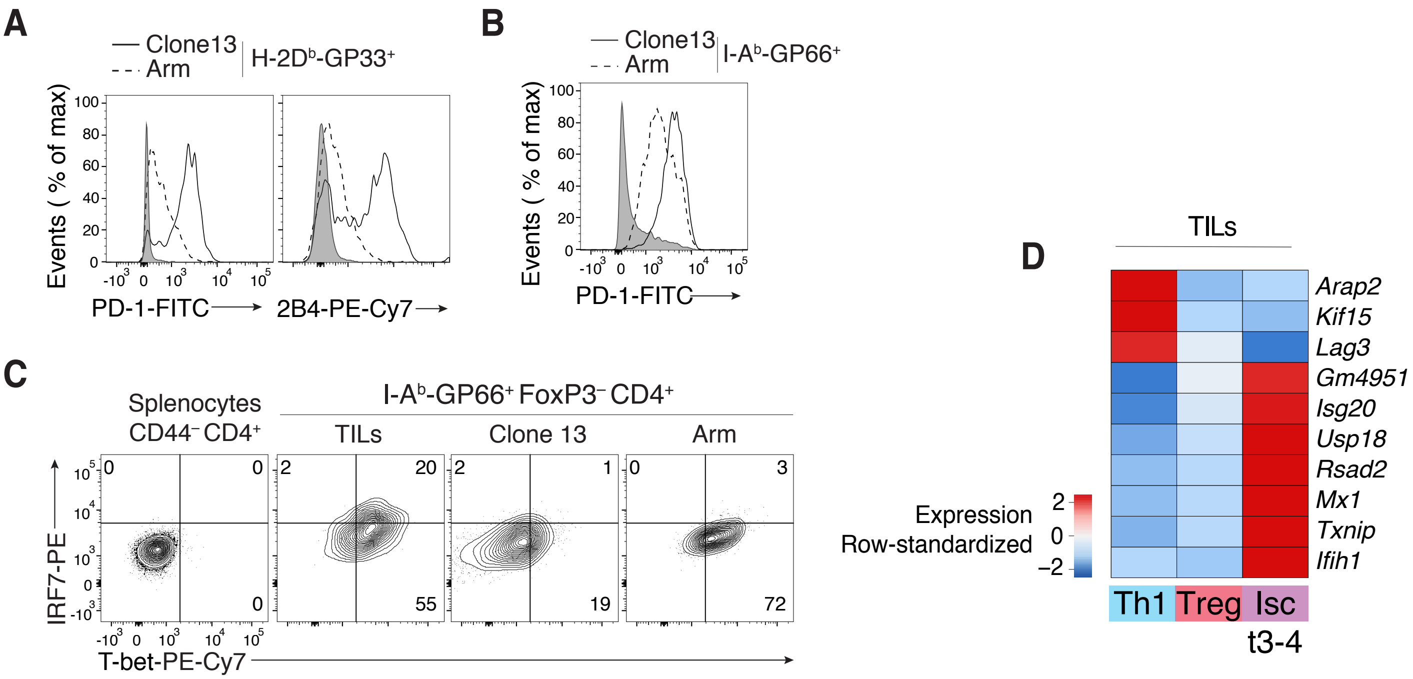
A
Clone13
Arm
H-2Db-GP33+
Events (% of max)
100
80
60
40
20
0
-10^3 0 10^3 10^4 10^5
PD-1-FITC
2B4-PE-Cy7
B
Clone13
Arm
I-Ab-GP66+
Events (% of max)
PD-1-FITC
C
Splenocytes
CD44– CD4+
I-Ab-GP66+ FoxP3– CD4+
TILs
Clone 13
Arm
IRF7-PE
T-bet-PE-Cy7
0
0
0
2
20
55
2
1
19
0
3
72
D
TILs
Expression
Row-standardized
2
0
−2
Arap2
Kif15
Lag3
Gm4951
Isg20
Usp18
Rsad2
Mx1
Txnip
Ifih1
Th1
Treg
Isc
t3-4

Figure S5. Characterization of the exhaustion status in clone 13 cells and TILs, Related to Figure 5.

(A) Overlaid protein expression of PD-1 or 2B4 in $GP33^+$ $CD8^+$ splenocytes from Arm- or Clone 13-infected mice.

(B) Overlaid protein expression of PD-1 in $GP66^+$ $CD4^+$ splenocytes from Arm- or Clone 13-infected mice. Grey shaded trace (A, B) is control from tumor-free mice.

(C) Flow cytometry contour plots of Tbet vs. IRF7 in Foxp3 $GP66^+$ TILs, Clone 13, or Arm cells, and in naive $CD4^+$ splenocytes from tumor-free control mice. (A-C) Data is representative of 10 mice analyzed in two experiments.

(D) Heatmap shows row-standardized expression of selected exhaustion genes across TIL Th1, Treg and Isc clusters (respectively clusters t1-2, t6-7 and t3-4 as shown in Fig. 1A).

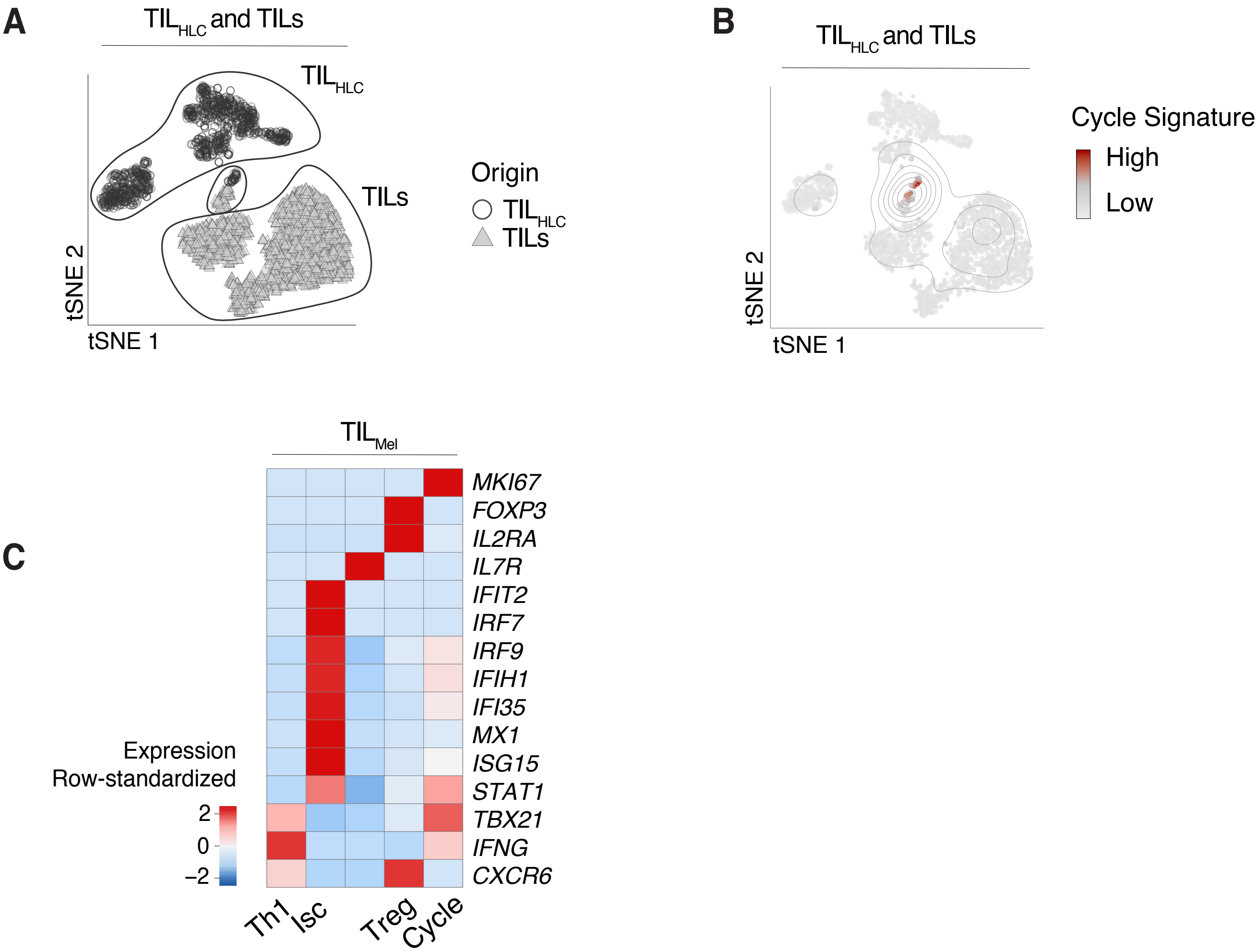
A
TILHLC and TILs
TILHLC
TILs
Origin
TILHLC
TILs
tSNE 2
tSNE 1
B
TILHLC and TILs
Cycle Signature
High
Low
tSNE 2
tSNE 1
C
TILMel
MKI67
FOXP3
IL2RA
IL7R
IFIT2
IRF7
IRF9
IFIH1
IFI35
MX1
ISG15
STAT1
TBX21
IFNG
CXCR6
Expression
Row-standardized
2
0
−2
Th1
Isc
Treg
Cycle

Figure S6. Correspondence to human data and dysfunction gene signatures, Related to Figure 6.

(A-B) Analysis of TIL_{HLC} and TILs (as defined in text). **(A)** tSNE plots show cells grey-shaded by origin. **(B)** tSNE plots show cells color-coded by cell cycle signature activation level.

(C) Analysis of TIL_{Mel} (as defined in text). Heatmap shows row-standardized expression of selected TIL characteristic genes across TIL_{Mel} clusters.
