# Supplementary material for: Single-Cell Profiling Defines Transcriptomic Signatures Specific to Tumor-Reactive versus Virus-Responsive CD4+ T Cells
Source: Cell Rep. Author manuscript; Available in PMC 2019 Dec 27. (PMC6934378; doi:10.1016/j.celrep.2019.10.131)
Supplement: 8 [file NIHMS1545825-supplement-8.pdf]

Cell Reports

Article

Single-Cell Profiling Defines Transcriptomic Signatures Specific to Tumor-Reactive versus Virus-Responsive CD4$^+$ T Cells

Graphical Abstract

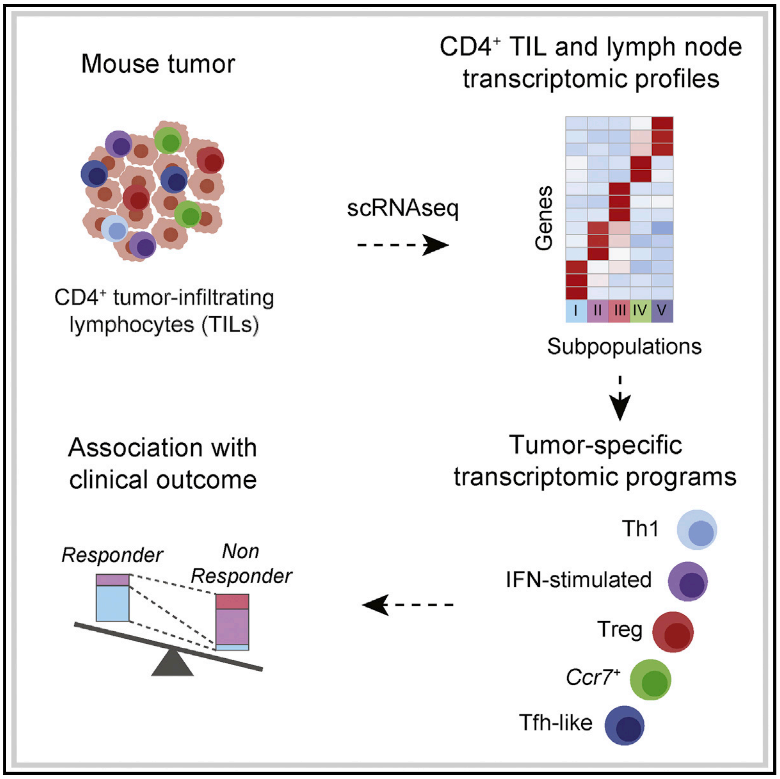

Highlights

- Single-cell RNA-seq analyzes antigen-specific tumor-infiltrating lymphocytes (TILs)
- CD4$^+$ TIL responses are highly heterogenous and distinct from anti-viral responses
- Th1-like TILs show evidence of type I interferon-driven signaling
- Interferon signature is negatively associated with human tumor response to therapy

Authors

Assaf Magen, Jia Nie, Thomas Ciucci, ..., Dorian B. McGavern, Sridhar Hannenhalli, Rémy Bosselut

Correspondence

remy.bosselut@nih.gov

In Brief

CD4$^+$ T cells contribute to immune responses to tumors, but their functional diversity has hampered their utilization in clinical settings. Magen et al. use single-cell RNA sequencing to dissect the heterogeneity of CD4$^+$ T cell responses to tumor antigens and reveal molecular divergences between anti-tumor and anti-viral responses.

Magen et al., 2019, Cell Reports *29*, 3019–3032
December 3, 2019
https://doi.org/10.1016/j.celrep.2019.10.131

CellPress

Cell Reports
Article

OPEN ACCESS
CellPress

Single-Cell Profiling Defines Transcriptomic Signatures Specific to Tumor-Reactive versus Virus-Responsive CD4+ T Cells

Assaf Magen,[1,2,8,9] Jia Nie,[1,9] Thomas Ciucci,[1] Samira Tamoutounour,[3] Yongmei Zhao,[4] Monika Mehta,[5] Bao Tran,[5] Dorian B. McGavern,[6] Sridhar Hannenhalli,[3,7,10] and Rémy Bosselut[1,10,11,*]
[1]Laboratory of Immune Cell Biology, Center for Cancer Research, National Cancer Institute, NIH, Bethesda, MD, USA
[2]Center for Bioinformatics and Computational Biology, University of Maryland, College Park, MD, USA
[3]Metaorganism Immunology Section, Laboratory of Immune System Biology, National Institute of Allergy and Infectious Diseases, NIH, Bethesda, MD, USA
[4]Advanced Biomedical and Computational Science, Frederick National Laboratory for Cancer Research, Frederick, MD, USA
[5]NCI CCR Sequencing Facility, Frederick National Laboratory for Cancer Research, Frederick, MD, USA
[6]Viral Immunology and Intravital Imaging Section, National Institute of Neurological Disorders and Stroke, NIH, Bethesda, MD, USA
[7]Present address: Cancer Data Science Laboratory, National Cancer Institute, National Institutes of Health, Bethesda, MD, USA
[8]Present address: Department of Oncological Sciences, Icahn School of Medicine at Mount Sinai, New York, NY 10029, USA
[9]These authors contributed equally
[10]These authors contributed equally
[11]Lead Contact
*Correspondence: remy.bosselut@nih.gov
https://doi.org/10.1016/j.celrep.2019.10.131

SUMMARY

Most current tumor immunotherapy strategies leverage cytotoxic CD8+ T cells. Despite evidence for clinical potential of CD4+ tumor-infiltrating lymphocytes (TILs), their functional diversity limits our ability to harness their activity. Here, we use single-cell mRNA sequencing to analyze the response of tumor-specific CD4+ TILs and draining lymph node (dLN) T cells. Computational approaches to characterize subpopulations identify TIL transcriptomic patterns strikingly distinct from acute and chronic anti-viral responses and dominated by diversity among T-bet-expressing T helper type 1 (Th1)-like cells. In contrast, the dLN response includes T follicular helper (Tfh) cells but lacks Th1 cells. We identify a type I interferon-driven signature in Th1-like TILs and show that it is found in human cancers, in which it is negatively associated with response to checkpoint therapy. Our study provides a proof-of-concept methodology to characterize tumor-specific CD4+ T cell effector programs. Targeting these programs should help improve immunotherapy strategies.

INTRODUCTION

Immune responses have the potential to restrain cancer development, and most immunotherapy strategies aim to reinvigorate T cell function to unleash effective anti-tumor immune responses (Borst et al., 2018; Gajewski et al., 2013; Ribas and Wolchok, 2018; Rosenberg and Restifo, 2015; Wei et al., 2017). Cytotoxic CD8+ T lymphocytes are being exploited in clinical settings because of their ability to recognize tumor neo-antigens and kill cancer cells (Ott et al., 2017; Rosenberg and Restifo, 2015). However, effective anti-tumor immunity relies on a complex interplay between diverse lymphocyte subsets that remain poorly characterized. CD4+ T helper cells, which are essential for effective immune responses and control the balance between inflammation and immunosuppression (Bluestone et al., 2009; Borst et al., 2018; Sakaguchi et al., 2008; Zhu et al., 2010), have recently emerged as potential therapeutic targets (Aarntzen et al., 2013; Borst et al., 2018; Hunder et al., 2008; Malandro et al., 2016; Mumberg et al., 1999; Ott et al., 2017; Tran et al., 2014; Wei et al., 2017). CD4+ helper cells contribute to the priming of CD8+ T cells and to B cell functions in lymphoid organs (Ahrends et al., 2017; Borst et al., 2018; Crotty, 2015). CD4+ T helper type 1 (Th1) cells secrete the cytokine interferon (IFN)-γ and affect tumor growth by targeting the tumor microenvironment (TME), antigen presentation through major histocompatibility complex (MHC) class I and MHC class II, and other immune cells (Alspach et al., 2019; Beatty and Paterson, 2001; Bos and Sherman, 2010; Kammertoens et al., 2017; Qin and Blankenstein, 2000; Tian et al., 2017). Conversely, T helper type 2 (Th2) cells can promote tumor progression, whereas regulatory T (Treg) cells mediate immune tolerance, suppressing the function of other immune cells and thus preventing ongoing anti-tumor immunity (Chao and Savage, 2018; DeNardo et al., 2009; Tanaka and Sakaguchi, 2017).

Despite the anti-tumor potential of CD4+ T cells, disentangling their functional diversity has been the limiting factor for pre-clinical and clinical progress. Although several studies have assessed the transcriptome of Treg cells or their tumor reactivity (Ahmadzadeh et al., 2019; Chao and Savage, 2018; De Simone et al., 2016; Malchow et al., 2013; Plitas et al., 2016; Zhang et al., 2018; Zheng et al., 2017a), the functional diversity of conventional (non-Treg) tumor-infiltrating lymphocytes (TILs) has

This is an open access article under the CC BY license (http://creativecommons.org/licenses/by/4.0/).

remained poorly understood. Population studies have limited power at identifying new, and especially rare, functional cell states. Conventional single-cell approaches (e.g., flow or mass cytometry) overcome this obstacle but are necessarily restricted to hypothesis-based targets because of the number of parameters they can analyze. Furthermore, most previous studies, whether of human or experimental tumors, did not distinguish tumor antigen-specific from bystander CD4$^+$ T cells, even though bystanders may form most conventional (non-Treg) T cells in the TME (Ahmadzadeh et al., 2019; Azizi et al., 2018; Duhen et al., 2018; Sade-Feldman et al., 2018; Simoni et al., 2018; Zhang et al., 2018; Zheng et al., 2017a) and in draining lymphoid organs where immune responses are typically initiated.

To address these challenges, we applied the resolution of single-cell RNA sequencing (scRNA-seq) to a tractable experimental system assessing tumor-specific responses both in the tumor and in the lymphoid organs, and we designed computational analyses to identify transcriptomic similarities. Our analyses dissect the complexity of the CD4$^+$ T cell response to tumor antigens and identify broad transcriptomic divergences between anti-tumor and both acute and chronic anti-viral responses. Emphasizing the power of this approach, transcriptomic patterns identified in the present study are also found in CD4$^+$ T cells infiltrating human tumors and correlate with response to checkpoint therapy in human melanoma.

RESULTS

Tracking Tumor-Specific CD4$^+$ T Cells

We set up a tractable experimental system to study tumor antigen-specific CD4$^+$ T cells. We retrovirally expressed the lymphocytic choriomeningitis virus (LCMV) glycoprotein (GP) in colon adenocarcinoma MC38 cells, using a vector expressing mouse Thy1.1 as a reporter (Figure S1A). Subcutaneous injection of the resulting MC38-GP cells produced tumors, allowing analysis of immune responses by day 15 after injection. We tracked GP-specific CD4$^+$ T cells through their binding of tetramerized I-A^b MHC class II molecules associated with the GP-derived GP66 peptide (Matloubian et al., 1994). Such CD4$^+$ cells were found in the tumor and draining lymph node (dLN) of MC38-GP tumor-bearing mice but in neither non-draining LN (nLN) from MC38-GP mice nor mice carrying control MC38 tumors (Figure S1B). TILs and dLN also included small numbers of CD8$^+$ T cells specific for the GP-derived GP33 peptide complexed with H-2D^b MHC class I molecules (Figure S1C). As expected, these cells expressed the transcription factor T-bet (Figure S1D).

To study the CD4$^+$ T cell response to tumor antigens, we aimed to produce genome-wide single-cell mRNA expression profiles (scRNA-seq) in CD4$^+$ TILs and CD4$^+$ dLN cells. We sorted GP66-specific T cells from dLN cells, because these were the only dLN CD4$^+$ T cells for which tumor specificity could be ascertained. Among TILs, we noted that ~87% of GP66-specific CD4$^+$ T cells expressed programmed cell death 1 (PD-1), encoded by *Pdcd1* and a marker of antigenic stimulation (Agata et al., 1996), suggesting that it could serve as an indicator of tumor specificity (Figure S1E). Alternatively, we considered using CD39 to this end, because CD39 marks CD8$^+$ TILs specific to tumor antigens (Duhen et al., 2018; Simoni et al., 2018). However, whereas CD39 expression was detected on most Foxp3$^+$ (Treg) GP66-specific TILs, it was low or undetectable on their Foxp3$^-$ counterparts, most of which were PD-1^{hi} (Figure S1F); this is consistent with previous reports that CD39 is preferentially expressed in Treg cells among CD4$^+$ T cells (Bono et al., 2015). Thus, to obtain a broad representation of antigen-specific TILs, not limited to GP-specific cells, we used PD-1 expression as a surrogate for tumor antigen specificity and purified tumor CD4$^+$CD44^{hi}PD-1$^+$ T cells (PD-1^{hi} TIL) for scRNA-seq. We verified critical conclusions of the scRNA-seq analyses by flow cytometry, comparing GP66-specific and PD-1^{hi} TILs.

Tumor-Responsive CD4$^+$ T Cells Are Highly Diverse

We captured GP66-specific dLN and PD-1^{hi} TIL CD4$^+$ cells using the 10x Chromium scRNA-seq technology (Zheng et al., 2017b); in addition, we captured GP66-specific spleen CD4$^+$ T cells from LCMV (Armstrong [Arm] strain)-infected mice (Matloubian et al., 1994) as a technical and biological reference (Figure S1G, called Arm cells here). After excluding cells of low sequencing quality (low number of detected genes), potential doublets, and B cell contaminants, we performed a first series of analyses on 566 dLN, 730 TIL, and 2,163 Arm CD4$^+$ T cells (Table S1).

We defined groups of cells sharing similar transcriptomic profiles using Phenograph clustering (Levine et al., 2015). Consistent with previous studies (Ciucci et al., 2019), Arm cells segregated into T follicular helper cells (Tfh cells, providing help to B cells) and Th1 cells, among other subsets (Figure S2A). Tfh cells expressed *Tcf7* (encoding the transcription factor Tcf1), *Cxcr5*, and *Bcl6*, whereas Th1 cells expressed *Tbx21* (encoding the transcription factor T-bet), *Ifng* (IFNγ), and *Cxcr6*. Low-resolution clustering identified 5 groups of TILs and dLN cells (Figure S2B). Group I had features of Th1 cells, whereas group II differed by lower expression of *Tbx21* and *Ifng* and expressed the chemokine receptor *Cxcr3* and the transcription factor *Irf7*. Group III expressed genes typical of Treg cells, including *Foxp3* and *Il2ra*, encoding CD25 (IL-2Rα). Group IV expressed *Ccr7*, which preferentially marks memory cell precursors at the early phase of the immune response (Ciucci et al., 2019; Pepper and Jenkins, 2011), whereas group V expressed Tfh cell genes, including *Bcl6* and *Cxcr5*.

To further dissect these populations, we developed a user-independent, data-driven approach to increase clustering resolution while controlling for false discovery. Applying such high-resolution clustering separately to TILs and dLN cells, we identified 15 clusters (TIL clusters t1–7 and dLN clusters n1–8), refining the original five main groups (Figure 1A). Revealing unexpected diversity among Th1-like TILs, groups I and II resolved into 5 subpopulations, including a distinct cluster (t5) expressing higher levels of *Il7r* (encoding IL-7Rα) and lower levels of *Tbx21* and *Ifng*. Only cluster group III (Treg cells) included both TIL and dLN cells, which expressed variable levels of *Tbx21*. Groups IV and V, the bulk of dLN cells, resolved into 5 and 2 clusters, respectively. Consistent with these results, flow cytometric analysis showed that most dLN cells expressed low or undetectable amounts of T-bet, the product of *Tbx21*; in contrast, most TILs expressed T-bet, even if at various levels (Figures 1B and 1C).

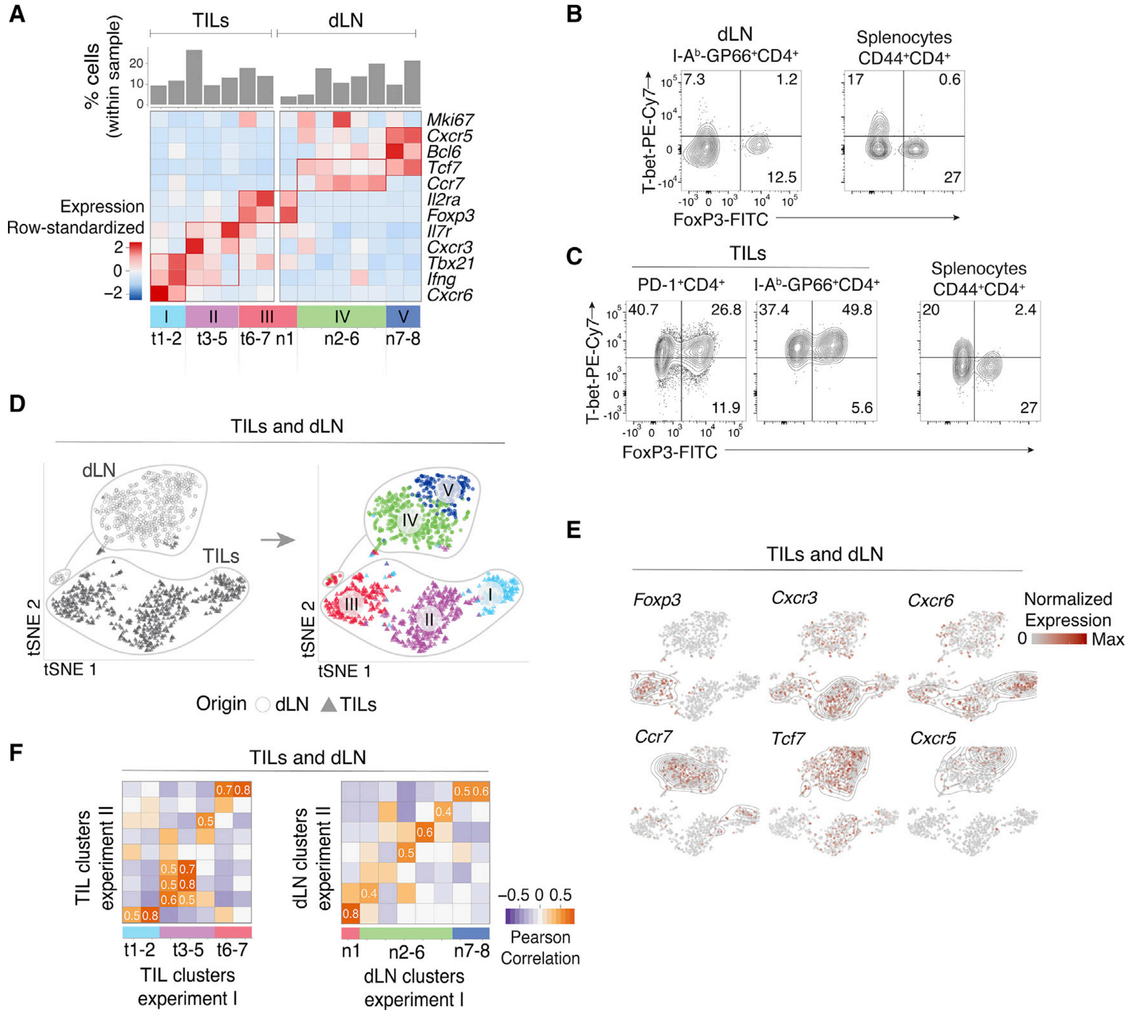

Figure 1. Characterization of CD4$^+$ TIL, dLN, and Arm Transcriptomes by scRNA-Seq

(A–D) TILs and dLN cells from wild-type (WT) mice at day 14 after MC38-GP injection analyzed by scRNA-seq and flow cytometry.

(A) Heatmap shows row-standardized expression of selected genes across TIL and dLN clusters. Bar plot indicates the percentage of cells in each cluster relative to the total TIL or dLN cell number.

(B) Flow cytometry contour plots of Foxp3 versus T-bet in CD44^{hi} GP66$^+$ dLN cells (left) and in CD44^{hi}CD4$^+$ splenocytes from tumor-free control mice (right).

(C) Flow cytometry contour plots of Foxp3 versus T-bet in PD-1$^+$ and GP66$^+$ TILs (left) and in CD44^{hi} CD4$^+$ splenocytes from tumor-free control mice (right).

(B and C) Data representative from 18 tumor-bearing mice analyzed in four separate experiments.

(D) t-SNE display of TILs and dLN cells, shaded gray by tissue origin (left) or color coded by main group (right, as defined in A).

(E) t-SNE (TIL and dLN cell positioning as shown in B) display of normalized expression levels of selected genes.

(F) Heatmap shows Pearson correlation between cluster fold change vectors (as defined in the text) across the two replicate experiments for TILs (left) and dLN cells (right).

See also Figures S1 and S2 and Tables S1 and S6.

To support these observations, we analyzed pooled TILs and dLN cells by t-Distributed Stochastic Neighbor Embedding (t-SNE), a dimensionality reduction approach that positions cells on a two-dimensional grid based on transcriptomic similarity (van der Maaten and Hinton, 2008). Although performed on the pooled populations, t-SNE recapitulated the minimal overlap between TIL and dLN transcriptomic patterns (Figure 1D, left), irrespective of parameter selection (Figure S2C) and even after controlling for potential confounders (Figures S2D and S2F–S2H; STAR Methods). Cluster groups I–V segregated from each other when projected on the t-SNE plot (Figure 1D, right). Overlay of gene expression confirmed co-localization of cells expressing cluster-characteristic genes (Figure 1E).

To verify the robustness of these observations, we analyzed an additional biological replicate consisting of 1,123 TILs, 675 dLN GP66-specific cells, and 2,580 Arm cells captured from a separate set of animals (Figure S2E; Table S1). Because batch-specific effects can confound co-clustering from distinct experiments, we separately clustered cells from each replicate. To compare these clusters, we evaluated the correlation

between cluster-specific fold change vectors; these vectors, defined internally to each replicate, recorded the expression of each gene in a cluster relative to all other clusters in that replicate. This strategy corrects for experiment-specific biases to allow effective comparison of cell subsets. We found significant inter-replicate matches for most clusters (Figure 1F), supporting the reproducibility of the underlying transcriptomic patterns. Thus, scRNA-seq analysis of tumor-specific CD4$^+$ T cells identifies an unsuspected diversity of transcriptomic programs in the TME and dLN.

Correlation Analyses Mitigate Tissue-Context-Specific Factors

Comparison of TILs, dLN cells, and Arm cells showed little overlap, including between TILs and dLN cells (Figure S3A, left). Thus, we considered that the impact of tissue of origin could be the primary driver of clustering and mask commonalities in effector programs. Indeed, most TIL subpopulations had attributes of tissue residency, including low *S1pr1* and *Klf2* expression and high *Cd69* expression, contrasting with Arm and most tumor dLN clusters (Figure 2) (Mackay and Kallies, 2017). Only group III Treg cells, and separately cells undergoing cell cycle, clustered together regardless of origin (Figure S3A, right). This prompted us to search for potential underlying similarities among these disparate transcriptomic patterns. We found that data integration approaches designed to uncover similarities across experimental conditions could not overcome the separation resulting from biological context (Figure S3B) and could miss functionally relevant differences (e.g., between *Foxp3*$^+$ and *Foxp3*$^-$ TILs) (Figure S3C) (Butler et al., 2018). Thus, we considered the correlation analysis used earlier for cluster matching, where Pearson correlation coefficients quantify similarities between cluster-specific fold change vectors. This analysis distributed the 40 reproducible clusters (out of 47 from all experiments) into 6 meta-clusters (with manual curation attaching meta-cluster 1^b to 1^a), of which four meta-clusters (meta-clusters 1, 3, 5, and 6) contained cells of more than one tissue context (Figure 3A; Table S1). Thus, the correlation analysis established relatedness among transcriptomic patterns identified by conventional clustering.

Characterizing Transcriptomic Similarities

We further characterized the meta-clusters by identifying their defining overexpressed genes. In addition to *Foxp3* and *Il2ra*, genes driving meta-cluster 3 (Treg cell group III) included *Ikzf2*, *Tnfrsf4*, encoding Ox40, and *Icos*, the latter of which we verified by flow cytometry (Figures 2, 3A, S3D, and S3F). In contrast, *Gzmb* (encoding the cytotoxic molecule granzyme B) and *Lag3* were overexpressed in TIL Treg cells relative to dLN Treg cells (and to *Foxp3*$^-$ TIL subsets) (Figures S3D–S3F). Thus, the similarity analysis both confirmed the shared Treg circuitry across TILs and dLN and identified TIL-specific *Gzmb* cytotoxic gene expression in TIL Treg cells.

Contrasting with the Treg clusters, the correlation analysis failed to detect similarities among three other groups characterized by heterogeneous *Tbx21* levels and distributed into meta-clusters 2 (TIL group II t3-4), 4 (Arm cells), and 6 (TIL group I t1-2) (Figure 3A). The two TIL meta-clusters showed multiple differences relative to Arm-responsive Th1 cells, including higher expression of *Il12rb*, *Il7r*, and *Il10ra* and distinct patterns of transcription factor, chemokine, and chemokine receptor expression (Figure 2). TIL group I t1-2 clusters (Th1 hereafter) specifically expressed *Lag3* and killer cell lectin (Klr) genes (Figures 3B, right, 3C, and S3G), characteristic of terminally differentiated effector cells (Joshi and Kaech, 2008), and differed from Arm Th1 by the expression of multiple activation molecules (Figure S3H). Accordingly, flow cytometry verified expression of CD94 and NKG2A (encoded by *Klrd1* and *Klrc1*, respectively) in a subset of GP66-specific TILs, whereas no expression was detected among GP66-specific Arm or dLN cells (Figure 3D, top). TIL group II t3-4 cells differed from the other T-bet-expressing cells by high expression of multiple type I IFN-induced genes, including transcription factors *Irf7* and *Irf9* (Figures 3B, left, 3C, and S3G). Accordingly, we designated group II t3-4 as IFN-stimulated cell (Isc) clusters. Consistent with the scRNA-seq analysis, flow cytometry detected IRF7 protein expression among GP66-specific TILs, but not Arm-responding CD4$^+$ T cells (Figure 3D, bottom); furthermore, flow cytometry distinguished the IRF7^{hi} (Isc) from NKG2A$^+$ (Th1) TIL subsets (Figure 3D). We noted that NKG2A$^+$ cells had higher expression of T-bet protein than other Foxp3$^-$ TILs (Figure 3E). Thus, because T-bet normally represses genes induced by type I IFN (Iwata et al., 2017), we verified co-expression of T-bet and IRF7 by intra-cellular staining and flow cytometry (Figure 3F). Consistent with high expression of the *Ifng* gene by Th1 TILs, NKG2A$^+$ TILs produced IFNγ protein when stimulated, unlike NKG2A$^-$ TILs (Figure 3G). Th1 TILs did not express the natural killer (NK) T cell-specific transcription factor PLZF, indicating they were not NK T cells (Figure S3I).

Compared with Isc, Th1 clusters had higher expression of *Bhlhe40*, encoding a transcription factor controlling inflammatory Th1 fate determination (Figures 2 and S3G) (Sun et al., 2001; Yu et al., 2018). A recent study of human colon cancer identified a CD4$^+$ TIL Th1 subset with elevated *Bhlhe40* expression (Zhang et al., 2018). This subset is clonally expanded in tumors with microsatellite instability, suggesting specificity for tumor antigens. The mouse Th1 TILs identified in our study had higher expression of 40 genes from the human colon TIL Th1 signature, including *Bhlhe40* and *Lag3* (Table S2), with significant ($p = 0.001$) skewing toward this signature detected by gene set enrichment analysis (GSEA) (Subramanian et al., 2005). However, mouse Th1 TILs lacked expression of other components of the human signature, including *Gzmb* and *Irf7*, suggesting that the impact of *Bhlhe40* expression on TIL transcriptomes is partly context specific.

Meta-cluster 6 unexpectedly associated Th1 TILs and a dLN *Ccr7*$^+$ cluster (the group IV n5 cluster) (Figure 3A), suggesting a potential link between TILs and dLN cells. The association was driven by transcriptional regulators *Bhlhe40* and *Id2* and tumor necrosis factor (TNF) superfamily members *Tnfsf8* (encoding CD30L) and *Tnfsf11* (RANKL) (Figures 2 and 4A). The potential connection between *Ccr7*$^+$ dLN cells and Th1 TILs was specific to *Ccr7*$^+$ cluster n5, which segregated from n6 and other dLN subsets (Tfh and Treg cells) based partly on higher expression of *Cd200* (Figure 4B). Flow cytometry identified a corresponding CD200^{hi} subset among Cxcr5^{lo} Ccr7$^+$, but not Cxcr5$^+$ Ccr7$^-$

OPEN
ACCESS
CellPress

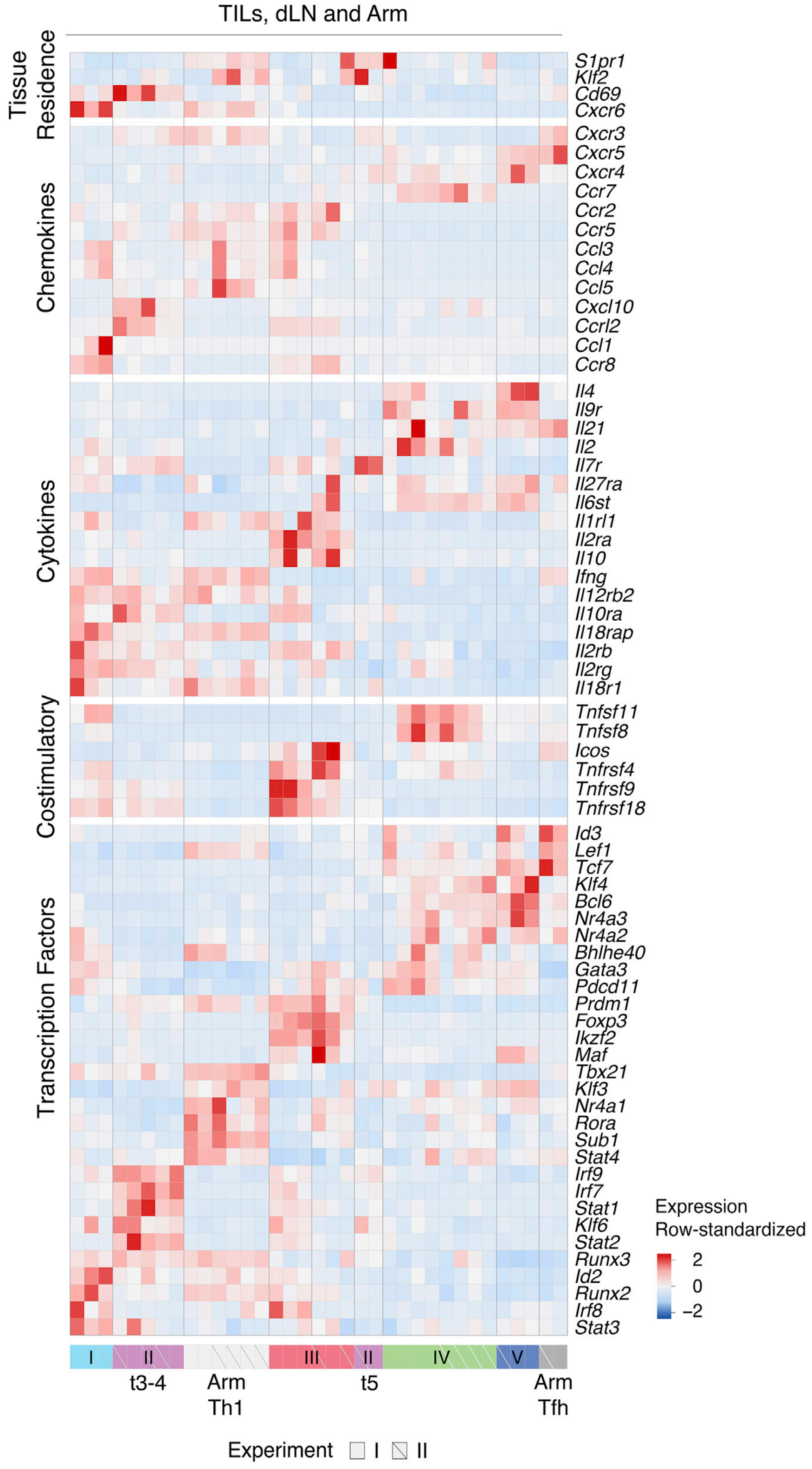

Figure 2. Transcriptomic Patterns of TILs, dLN Cells, and Arm Cells

TILs, dLN cells, and Arm cells from replicate experiments I and II analyzed by scRNA-seq. Heatmap shows row-standardized expression of selected genes across clusters. Group II (purple) t5 separated into a distinct component from t3-4 (as defined in the text). Of note, high-level expression of T-bet and other genes in Arm cells (included in this dataset), reduces the *Z* score (row normalized) expression value for such genes in TILs or dLN cells, accounting for their apparent lower relative expression compared with that in Figures 1A and S2B.

See also Figure S2 and Table S2.

OPEN ACCESS
CellPress

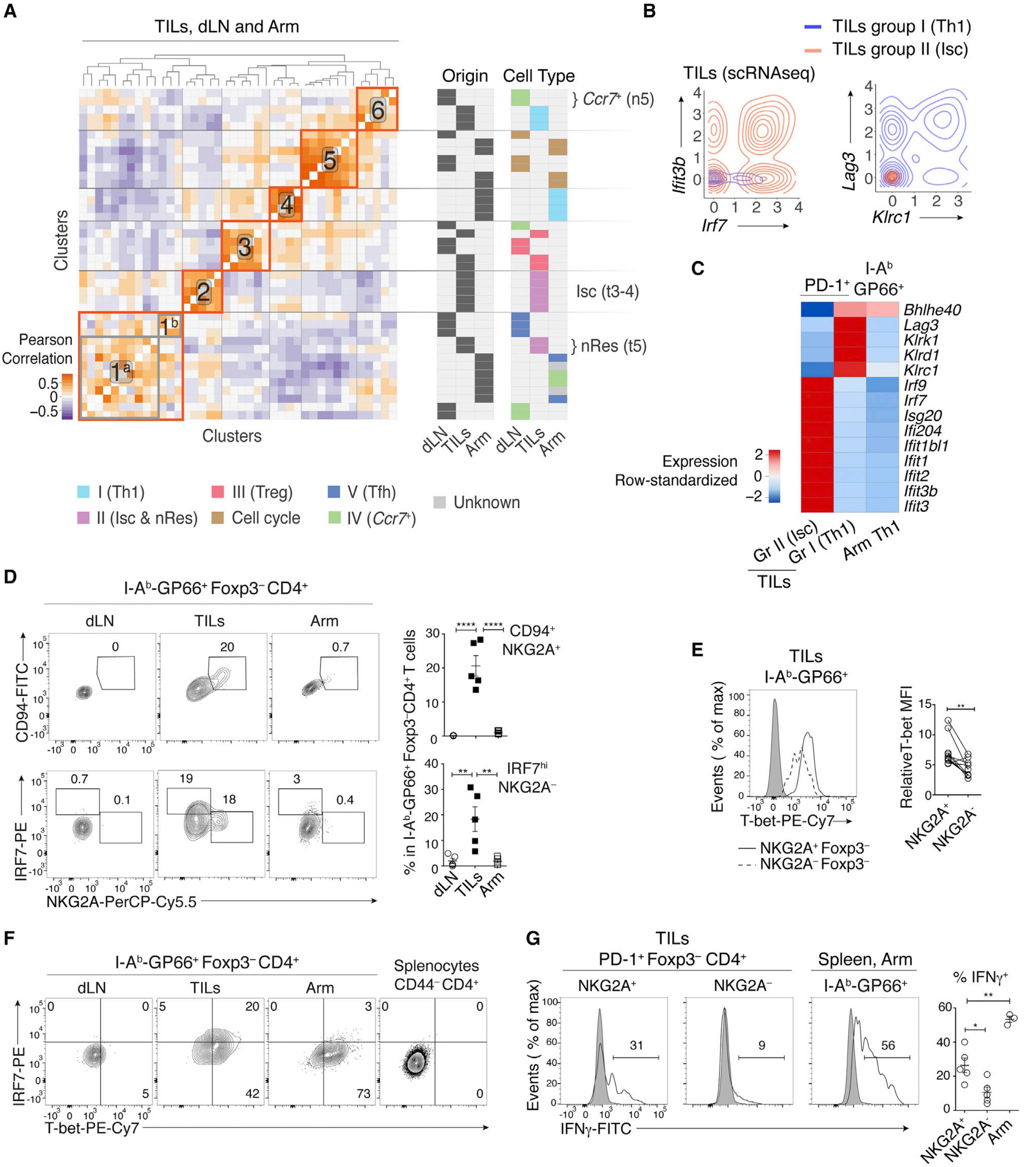

Figure 3. Th1-like Transcriptomic Patterns

(A) Heatmap defines meta-clusters based on Pearson correlation among TIL, dLN, and Arm cluster fold change vectors (as defined in the text) (left). Tables show tissue origin and cell-type color code per cluster (right).

(B and C) Comparison of TIL Th1 and Isc (clusters t1-2 and t3-4, respectively, as shown in Figure 1A), as well as Arm Th1 (as shown in Figures 2 and S2A).

(B) Contour plots of Th1 (orange) and Isc (blue) TIL distribution according to scRNA-seq-detected normalized expression of *Irf7* versus *Ifit3b* (left) and *Klrc1* versus *Lag3* (right).

(C) Heatmap shows row-standardized expression of differentially expressed genes across TIL group II Isc, TIL group I Th1, and Arm Th1.

(legend continued on next page)

(Tfh), GP66-specific cells (Figures 4C, S4A, and S4B). dLN *Ccr7*$^{+}$ clusters n5-6 shared features with central memory precursor CD4^{+} T cells (Tcmp cells) identified in Arm infection (Ciucci et al., 2019) (Table S2). This includes expression of *Tcf7*, a transcription factor important to prevent T cell terminal differentiation and for CD8^{+} T cell responsiveness to PD-1 blockade (Brummelman et al., 2018; Gattinoni et al., 2009; Im et al., 2016; Jeannet et al., 2010; Kurtulus et al., 2019; Nish et al., 2017; Siddiqui et al., 2019; Zhou et al., 2010). However, the correspondence between the MC38-GP dLN *Ccr7*$^{+}$ clusters and the Arm Tcmp signature was only partial (Table S2).

Meta-cluster 1 consisted of Arm Tfh clusters and dLN group V Tfh clusters (Figure 3A). We verified that the abundance of dLN Tfh cells was similar in mice carrying MC38-GP and MC38 tumors (Figure S4C), indicating that this response is not a consequence of GP expression. Flow cytometric analysis confirmed key Tfh attributes in dLN and Arm cells, including Bcl6 expression (Figures 4C, 4D, and S4A), although dLN Tfh cells differed from Arm-responsive Tfh cells by lower expression of *Icos* and the upregulation of the transcription factor *Maf* (Figures 2, 4E, and S4D). Unexpectedly, meta-cluster 1 associated the dLN and Arm Tfh clusters with TIL group II cluster t5, characterized by *Il7r* expression (Figures 1A and 3A), based partly on slightly higher expression of *Tcf7* (1.6-fold) relative to other TIL subpopulations (Figure 4F). Flow cytometric analysis confirmed the presence of GP66-specific IL-7R^{+} TILs (Figure 4G). In addition, the *Tcf7*^{int} t5 cluster showed expression of the transcription factor *Klf2* and its downstream target Sphingosine-1-phosphate receptor 1 (*S1pr1*, Figures 2 and 4F). This indicated the retention of a cell-trafficking transcriptional program (Carlson et al., 2006) and contrasted with the IFN-driven Isc TILs. Thus, we designated cluster t5 of group II TILs as putative non-resident cells (nRes hereafter).

To further delineate the relationships between cell clusters, we used reversed graph embedding (Trapnell et al., 2014), which has been used to estimate progression through transcriptomic states. This placed the dLN Tfh and TIL Th1 and Isc at the end of an inferred path (Figure 4H), nRes TILs in the middle of the continuum, and *Ccr7*$^{+}$ dLN cells between Tfh and nRes. These analyses, combined with the similarities described by meta-clustering, support the notion that the tumor-responsive CD4^{+} T cell response may be characterized as a transcriptomic continuum; they confirm the transcriptomic distance between Th1 and Isc TILs, even though both subsets express T-bet, the Th1-defining factor.

TIL Subpopulation-Specific Dysfunction Gene Programs

We reasoned that expression of a dysfunction-exhaustion program (Thommen and Schumacher, 2018; Wherry and Kurachi, 2015) may account for the limited relatedness between Arm and TIL Th1 cells, because TILs processed for scRNA-seq analysis expressed the exhaustion marker PD-1 and multiple genes associated with T cell exhaustion dysfunction (Figure 5A). To address this issue, we used flow cytometry to directly compare GP66-specific TILs from MC38-GP tumors to GP66-specific CD4^{+} T harvested 21 days after inoculation with the clone 13 strain of LCMV (clone 13 hereafter). This strain establishes chronic infection in wild-type mice (Oldstone, 2002), resulting in typical dysfunctional CD4^{+} and CD8^{+} T cell responses (Crawford et al., 2014). Most clone 13-responding CD8^{+} T cells expressed PD-1 and the surface receptor 2B4 (Figure S5A), characteristic of the dysfunction-exhaustion status of cells responding to persistent antigenic stimulation. Accordingly, PD-1 was expressed on most clone 13-responding spleen CD4^{+} T cells (Figure S5B), unlike among Arm-responding CD4^{+} T cells, in which PD-1 expression was specific to Cxcr5^{hi} Tfh cells (Figure 4D). Expression of PD-1 in GP66-specific TILs was similar to that in clone 13-responding cells (Figure 5B) and higher than in dLN GP66-specific cells (of which only the Cxcr5^{+} subset was PD-1^{hi}, Figure 4D). However, clone 13-responding CD4^{+} T cells failed to express key members of the TIL Th1 (CD94 and NKG2A) and Isc (IRF7) signatures (Figure 5C). Of note, clone 13-responding cells expressed lower amounts of T-bet compared with Arm- or MC38-GP-specific cells (Figure S5C). We conclude from these observations that the Th1 and Isc signatures of GP66-specific TILs are distinct from the dysfunction state generated by persistent antigen exposure.

Nonetheless, since CD4^{+} TILs expressed exhaustion marks (Figure 5A), we assessed the impact of exhaustion on TIL subpopulations. We defined TIL Th1, Isc, nRes, and Treg gene signatures as the genes preferentially expressed in each subpopulation relative to all other TILs (Table S3). We found a significant overlap between the multiple viral-response exhaustion gene signatures (Molecular Signatures Database [MSigDB]) (Liberzon et al., 2015) and the Th1 and Treg signatures (Table S4).

(D) (Left) Flow cytometry contour plots of NKG2A versus CD94 (top) or IRF7 (bottom) in Foxp3^{-}GP66^{+} dLN, TIL, and Arm cells. (Right) Percentage of NKG2A^{+}CD94^{+} cells (top) and IRF7^{hi} NKG2A^{-} cells (bottom) among Foxp3^{-}GP66^{+} CD4^{+} T cells; each symbol represents an individual mouse.

(E) Overlaid protein expression of T-bet in NKG2A^{+} and NKG2A^{-} Foxp3^{-}GP66^{+} TILs (left). The graph on the right summarizes quantification (mean fluorescence intensity, MFI) of T-bet in each subset, expressed relative to naive CD4^{+} splenocytes from tumor-free control mice. Each symbol represents an individual mouse; lines indicate pairing.

(F) Flow cytometry contour plots of T-bet versus IRF7 in Foxp3^{-}GP66^{+} dLN, TILs, and Arm cells; data from naive CD4^{+} splenocytes from tumor-free control mice is shown as a control (right plot).

(D–F) Each plot is representative from 10 tumor-bearing and 9 Arm-infected mice, analyzed in two separate experiments. Each symbol on summary graphs represents one mouse.

(G) (Left) Overlaid protein expression of IFNγ in NKG2A^{+} versus NKG2A^{-} TILs and Arm cells. Data are shown for Foxp3^{-}GP66^{+} cells (plain lines); expression on Foxp3^{+} cells is shown as a negative control (shaded gray). (Right) Graph shows the percentage of IFNγ^{+} cells out of NKG2A^{+} or NKG2A^{-} Foxp3^{-} TILs or of GP66^{+} Arm CD4^{+} T cells and summarizes a single experiment with 5 tumor-bearing and 3 Arm-infected mice. Data are representative of two such experiments, with 15 tumor-bearing and 5 Arm-infected mice. Each symbol on summary graphs represents one mouse.

Two-tailed unpaired (D and G) or paired (E) t test; *p < 0.05, **p < 0.01, and ****p < 0.0001.

See also Figure S3 and Table S2.

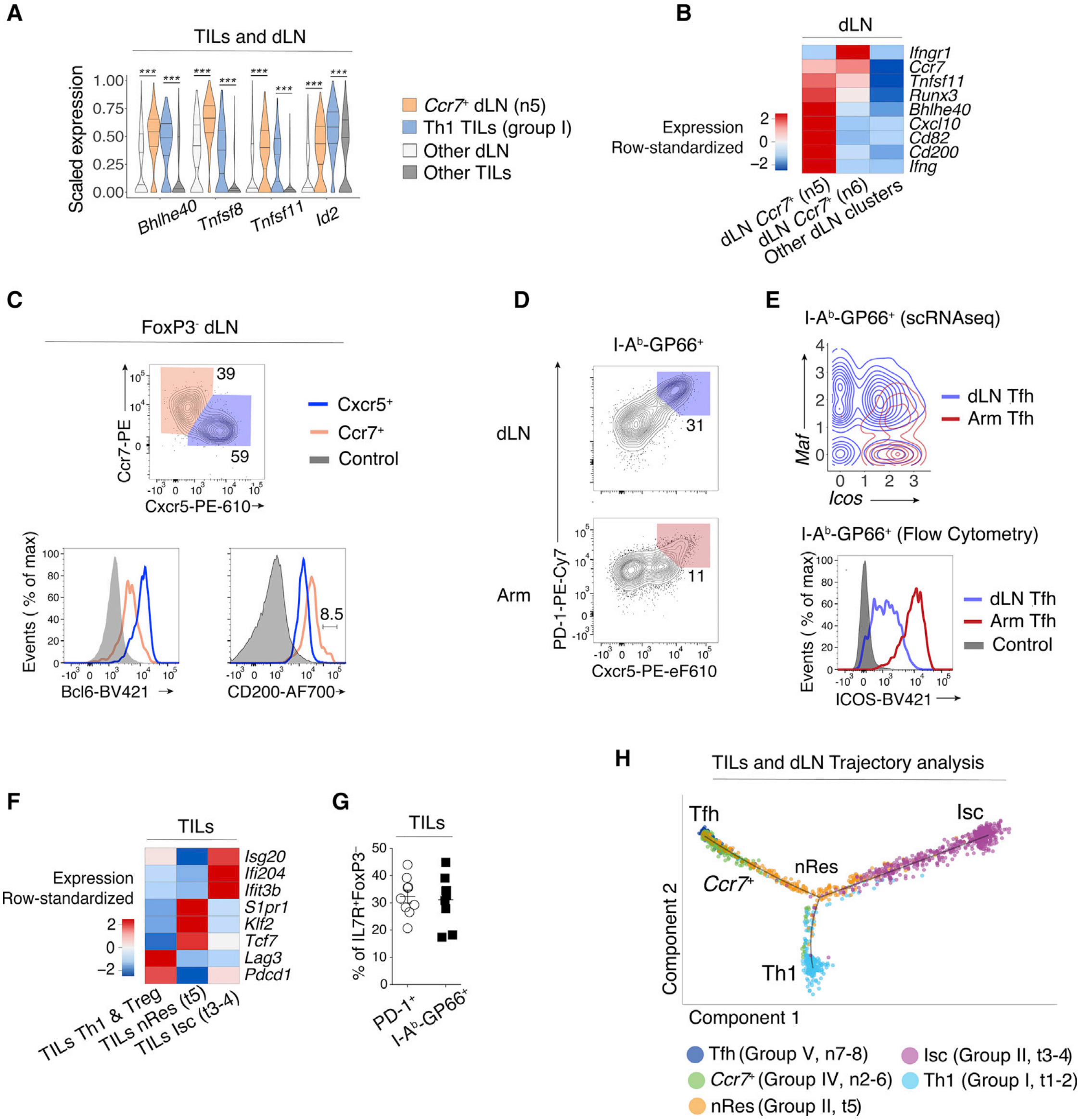

Figure 4. Transcriptomic Continuum between TIL and dLN Tumor-Reactive Cells

(A) Violin plots of differentially expressed genes across TIL group I Th1 and dLN group IV *Ccr7*$^+$ (clusters t1-2 and n5, respectively, as shown in Figure 1A), as well as all other TIL and dLN populations. Unpaired t-test; ***$p < 0.001$.

(B) Heatmap shows row-standardized expression of differentially expressed genes across dLN *Ccr7*$^+$ clusters (group IV n5-6) and other dLN clusters (Treg and Tfh clusters n1 and n7-8, respectively).

(C) Flow cytometry contour plots of Cxcr5 versus Ccr7 in Foxp3$^-$ dLN cells (top). Overlaid protein expression of Bcl6 and CD200 in Ccr7$^+$ and Cxcr5$^+$ dLN cells and naive CD4$^+$ splenocytes from tumor-free control mice (bottom). Data are representative of 17 mice analyzed in three experiments.

(D) Flow cytometry contour plots of Cxcr5 versus PD-1 in dLN and Arm cells. Data are representative of 10 mice analyzed in two experiments.

(E) Contour plot of dLN (red, clusters n7-8) and Arm (blue) Tfh cell distribution according to scRNA-seq-detected normalized expression of *Icos* versus *Maf* (top). Overlaid protein expression of ICOS in dLN and Arm PD-1$^+$Cxcr5$^+$ (Tfh) cells and naive CD4$^+$ splenocytes from tumor-free control mice (bottom).

(F) Heatmap shows row-standardized expression of differentially expressed genes across TIL Isc and nRes clusters (as defined in the text, group II t3-4 and t5, respectively) and all other TIL clusters (Th1 and Treg clusters t1-2 and t6-7, respectively).

(G) Percentage of IL7R$^+$Foxp3$^-$ cells out of total PD-1$^+$ or GP66$^+$ TILs. Nine mice analyzed in two experiments.

(H) Trajectory analysis of PD-1$^+$ TILs and GP66$^+$ dLN cells, indicating individual cells' assignment into a transcriptional continuum trajectory. nRes cluster (t5) is color coded orange in contrast to annotations in other figures.

See also Figure S4 and Table S2.

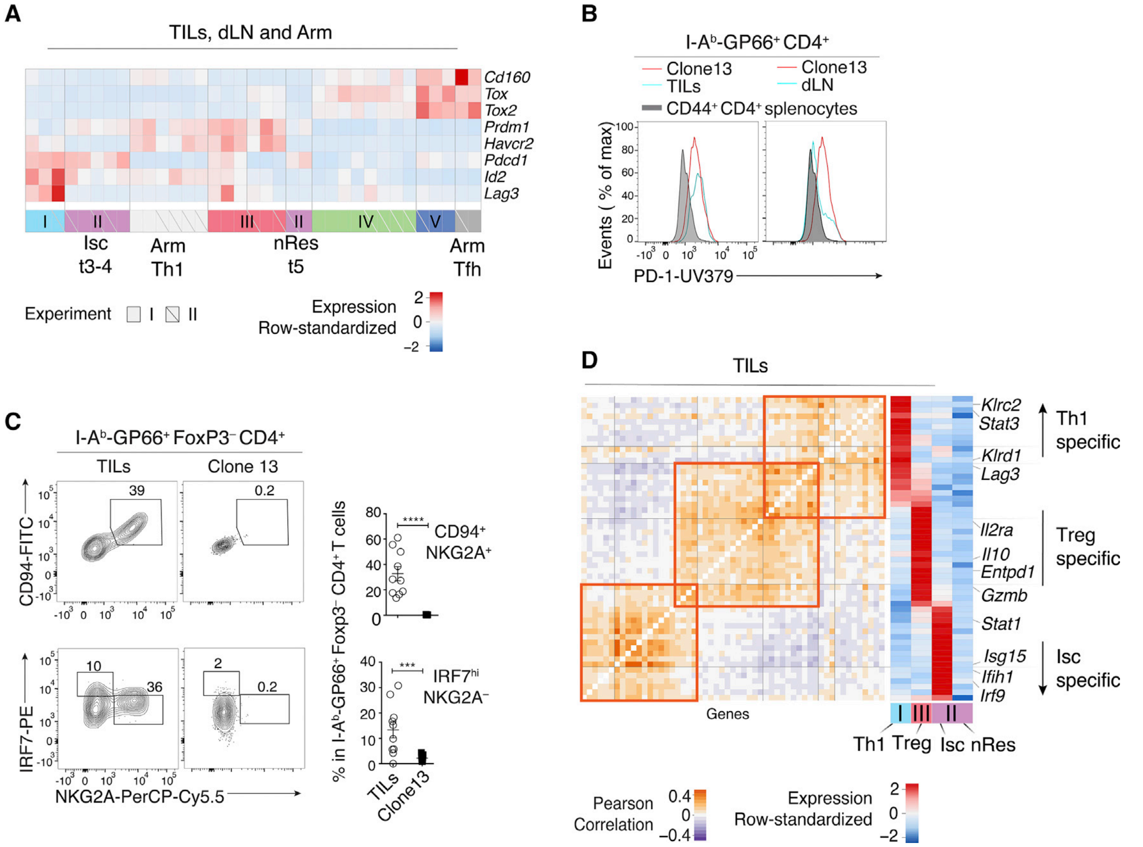

Figure 5. Dysfunction Transcriptomes of Th1, Isc, and Treg TILs

(A) Heatmap shows row-standardized expression of selected exhaustion genes across TIL, dLN, and Arm clusters from replicate experiments I and II.

(B) Overlaid protein expression of PD-1 in GP66$^+$ clone 13 (red trace) and GP66$^+$ TILs (left) or dLN cells (right) (cyan trace). Gray-shaded histograms show PD-1 expression on CD44$^+$CD4$^+$ splenocytes from tumor-free control mice.

(C) Flow cytometry contour plots of NKG2A versus CD94 (top) or IRF7 (bottom) in TILs and clone 13 Foxp3$^-$GP66$^+$ T cells. Graphs on the right summarize data from two experiments; each symbol represents one mouse. Two-tailed unpaired t test; ***p < 0.001 and ****p < 0.0001.

(B and C) Data are from 10 mice of each condition, analyzed on two separate experiments.

(D) Analysis of interleukin-27 (IL-27) signature genes overlapping with TIL subpopulation-characteristic genes. Heatmaps show Pearson correlation (left) and row-standardized expression of overlapping genes across TIL Th1, Treg, Isc, and nRes cells (clusters t1-2, t6-7, t3-4, and t5, respectively, as shown in Figure 1A) (right).

See also Figure S5 and Tables S3 and S4.

Separate analysis of a previously reported gene signature characterizing CD4$^+$ T cell dysfunction during chronic infection (Crawford et al., 2014) indicated a significant overlap with the Isc signature, but not with Th1 and Treg signatures (Figure S5D; Table S4). The latter result suggested heterogeneous expression of exhaustion genes among TIL subsets. We tested this possibility using a broader set of exhaustion genes shared across cancer and chronic infection (Chihara et al., 2018). Fifty-five genes from this set were also part of TIL Th1, Isc, or Treg signatures. However, the overlap was heterogeneous, identifying dysfunction programs specific to TIL subpopulations (Figure 5D; Table S4). We did not detect overlap between any dysfunction-exhaustion signature and nRes TILs (Figure 5D; Table S4). This is in line with these cells' residual expression of *Tcf7*, which in CD8$^+$ T cells marks cells with conserved responsiveness to checkpoint blockade (Brummelman et al., 2018; Im et al., 2016; Siddiqui et al., 2019; Wu et al., 2016).

The Isc IFN Signature Correlates with Poor Clinical Prognosis in Human Tumors

Finally, we examined whether MC38-GP TIL transcriptomic patterns were observed in human tumors. We analyzed published CD4$^+$ human liver cancer TIL (TIL$_{HLC}$) scRNA-seq data pooled across six treatment-naive patients (Zheng et al., 2017a). High-resolution clustering separated the TIL$_{HLC}$ cells into 11 clusters, which could be combined into groups displaying features of Th1, Isc (of which 36% are *PDCD1*$^+$), and Treg TILs and cells undergoing cell cycle (Figure 6A). Although pooled analysis of CD4$^+$ PD-1$^+$ TILs from MC38-GP tumors (TIL) with TIL$_{HLC}$ only identified similarities between cells undergoing cell cycle (Figures S6A and S6B), cluster correlation analysis indicated significant similarities between Treg cells, cell cycle, and Isc clusters from TIL versus TIL$_{HLC}$ (Figure 6B, top). We focused on the Isc pattern, which differed the most from previously reported Th1 and Treg transcriptomic profiles. We found significant overlap

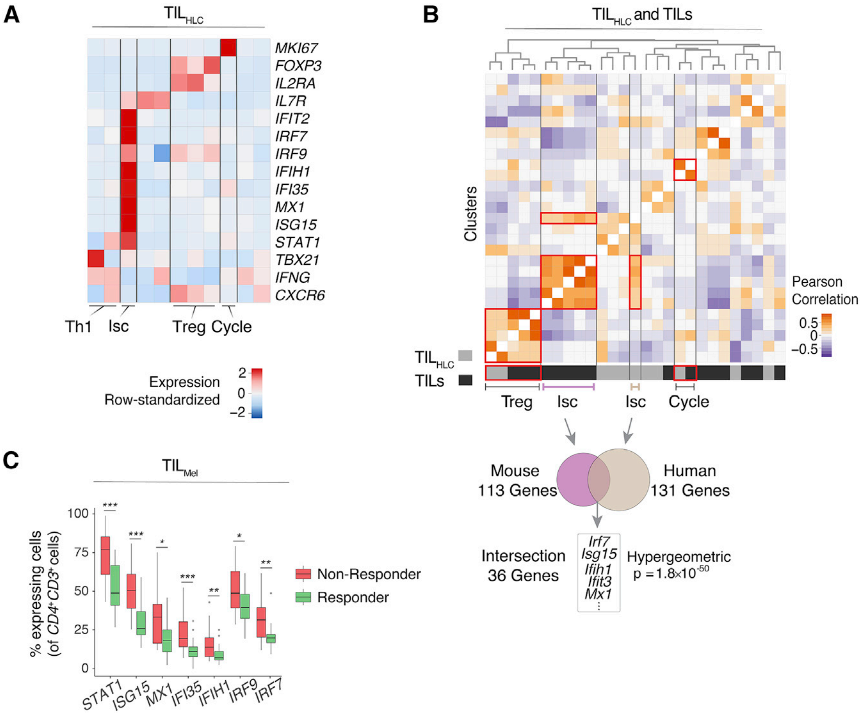

Figure 6. Correspondence to Human Data and Dysfunction Gene Signatures

(A) Analysis of human liver cancer TIL_{HLC}. Heatmap shows row-standardized expression of selected genes across TIL_{HLC} clusters.

(B) Heatmap defines meta-clusters based on Pearson correlation between TIL_{HLC} and MC38-GP TIL clusters (top). Overlap of genes characteristic of human liver TIL Isc cluster with mouse TIL Isc gene signature (bottom).

(C) Analysis of human melanoma TIL_{Mel}. Boxplots show the percentage of cells expressing selected IFN signaling-characteristic genes in *CD4⁺CD3⁺* cells across responding and non-responding lesions. Unpaired Wilcoxon test; $^{*}p < 0.05$, $^{**}p < 0.01$, and $^{***}p < 0.001$.

See also Figure S6 and Table S5.

of overexpression patterns between TIL Isc and their human counterpart, including type I IFN-induced genes and *Irf7* (Ikushima et al., 2013) (Figure 6B, bottom; Table S5). Thus, the Isc signature identified among mouse $CD4^+$ TILs is found in human tumors.

These finding were not unique to liver tumors, because analysis of $CD4^+CD3^+$ human melanoma TILs (TIL_{Mel}) across 48 lesions (Sade-Feldman et al., 2018) identified a cluster enriched in Isc-characteristic genes (of which 27% are *PDCD1*$^+$), among other populations (Figure S6C). To investigate the relationships between Isc transcriptomic program and clinical prognosis, we evaluated the association between expression in TIL_{Mel} of Isc signature genes (defined in MC38-GP TILs) and patient response to checkpoint therapy. Relative to responsive tumors, non-responsive tumors had significantly higher fractions of cells expressing Isc signature genes (49 of 108 genes, adjusted $p < 0.05$), including *Stat1*, *Irf7*, and *Irf9* (Figure 6C; Table S5). This indicated negative association between the Isc transcriptomic program and patient response to checkpoint therapy. Thus, the methods used in the present study identify transcriptomic programs shared by multiple tumor types and of potential prognostic significance.

DISCUSSION

In summary, using scRNA-seq and data-driven computational approaches, the present study identifies an unsuspected diversity among tumor-responding $CD4^+$ T cells. Although recent scRNA-seq studies had shed light on the Treg component of $CD4^+$ TILs (Ahmadzadeh et al., 2019; Azizi et al., 2018; Zhang et al., 2018; Zheng et al., 2017a), our study assessed the transcriptomes of both regulatory and conventional components, in the tumor itself, and in draining lymphoid organs. We identified transcriptomic patterns among these cells and found a heterogeneous distribution of exhaustion gene signatures among TIL subtypes, highlighting the need for extensive analyses of cell-specific effects of treatments targeting exhaustion genes.

One key objective of our study was to compare the transcriptome of $CD4^+$ T cells responding to tumors, whether in the tumor itself or in draining lymphoid organs, to that of cells responding to infection. To this end, we studied T cell responses to a viral antigen, LCMV GP, ectopically expressed in a mouse colon cancer cell line. This approach directly compares cells responding to the same antigen, expressed during viral infection or by tumor cells. In addition, because the Arm versus the clone 13 strains of LCMV, respectively, result in effective versus dysfunctional T cell responses, with chronic viral persistence after clone 13 strain infection, we could compare antigen-specific responses in each context with those against tumor cells. We considered that the potentially greater GP immunogenicity compared with that of spontaneously occurring tumor neo-antigens would skew GP-specific TILs toward specific transcriptomic patterns. Consequently, we extended our key conclusions beyond the limited set of TILs responding to the ectopic GP antigen, identifying PD-1 as a reliable marker of antigen-responsive cells and showing a broad correspondence between expression of key signature markers between PD-1^{hi} and GP-responsive TILs.

Even though most conventional ($Foxp3^-$) tumor-responsive TILs express the Th1-defining transcriptional regulator T-bet, our study identified transcriptomic patterns with unexpectedly little similarity to prototypical virus-responsive Th1 cells. Thus, conventional helper effector definitions, derived from studies of responses to infection, are potentially inaccurate descriptors of responses to tumors. The Th1-like transcriptome with marks of type I IFN stimulation, a driver of inflammation and immunosuppression in cancer (Snell et al., 2017), highlights this conclusion: it was observed among TILs, but not LCMV-responding cells, even though acute LCMV infection drives a strong type I

IFN innate immune response (Cousens et al., 1999). The transcriptomic definition of signatures had important functional correlates, because the type I IFN response signature was associated with lesser IFNγ production compared with cells expressing the Th1 signature. Future studies will determine whether any of these signatures, or those characteristic of tumor-responsive cells in the draining lymphoid organs, are associated with provision of help to $CD8^+$ T cells, which is essential for efficient anti-tumor responses (Ahrends et al., 2017; Bos and Sherman, 2010).

We considered the possibility that the distinct $CD4^+$ T cell responses to tumors versus infection resulted from differences in the kinetics of antigen exposure: transient during acute viral infection versus persistent exposure to tumor antigens. Expression of dysfunction-exhaustion genes, exemplified by PD-1, was a shared attribute of cells responding to tumor and chronic viral infection. However, the expression of type I IFN-responsive genes (Isc signature) was specific to tumor-responsive cells and not shared by anti-viral dysfunctional cells; the same was true of Klr-family receptors (Th1 signature). Our analyses point to the importance of these findings in the response to human cancer, because we could project the IFN-responsive transcriptomic pattern onto human tumors, overcoming potential sample disparity, and demonstrate its association with response to checkpoint therapy.

Investigating tumor-specific T cell responses in draining lymphoid organs revealed striking differences with TILs. The absence of Th1 cells from tumor dLN was unexpected and contrasted with infections, including with LCMV or with *Leishmania major*, a typical Th1-driving parasite with kinetics of clinical progression similar to that of experimental tumors and in which Th1 dLN cells are important contributors to the response (Belkaid et al., 2000). In contrast, the tumor elicited strong, tumor-specific Foxp3-negative Tfh-like responses in dLN. Similar populations of Tfh-like cells have been observed in human tumors (Crotty, 2019). Although Tfh differentiation may divert T cells from more efficient (e.g., IFNγ-producing) anti-tumor differentiation, it provides support for the tantalizing possibility that tumor-elicited B cell responses could be exploited against cancer (Carmi et al., 2015). It is also possible that this subset includes a stem cell-like component similar to the $Cxcr5^+$ $CD8^+$ dLN T cells that serve as targets for immunotherapy targeting PD-1 signaling (Im et al., 2016) or cells with similar properties in the TME (Siddiqui et al., 2019).

In conclusion, this study provides a high-resolution characterization of tumor-reactive $CD4^+$ T cell responses in lymphoid organs and the TME. We identify previously unrecognized transcriptomic patterns among tumor-specific T cells and provide an extensive mapping of the $CD4^+$ T cell immune response against cancer. We describe analytical approaches of broad applicability, including to clinical data, that combine high-resolution dissection of transcriptomic patterns and synthetic data integration to identify correspondences between apparently unrelated cell differentiation states.

STAR★METHODS

Detailed methods are provided in the online version of this paper and include the following:

- KEY RESOURCES TABLE
- LEAD CONTACT AND MATERIALS AVAILABILITY
- EXPERIMENTAL MODEL AND SUBJECT DETAILS
  - Mice
  - Cell Lines and Constructs
- METHOD DETAILS
  - LCMV Infection Model and Tumor Model
  - Cell Preparation and Flow Cytometry
  - Single-Cell RNA-Seq
- QUANTIFICATION AND STATISTICAL ANALYSIS
  - Experimental Data
  - scRNA-Seq Data Pre-processing
  - Transcriptomic Effects of TCR Engagement as a Result of GP66-Tetramer-Based Purification
  - Dimensionality Reduction
  - Gene Signature Activation Quantification
  - High-Resolution Clustering
  - Differential Expression Analysis and Population Matching
  - Robust Cluster Calling and Robust Population Comparisons
  - scRNaseq Contour Plots
  - scRNaseq Violin Plots
  - Reversed Graph Embedding
  - Gene Signature Definition
  - Correspondence to Human Data
  - Correspondence with External Gene Signatures
- DATA AND CODE AVAILABILITY

SUPPLEMENTAL INFORMATION

Supplemental Information can be found online at https://doi.org/10.1016/j.celrep.2019.10.131.

ACKNOWLEDGMENTS

We thank Melanie S. Vacchio for cell sorting; Mariah Balmaceno-Criss and Qi Xiao for animal genotyping; Jack Greiner for the MC38 cell line; the NIH tetramer facility for reagents; the CCR Flow Cytometry Core for expert assistance; Yasmine Belkaid, Avinash Bhandoola, and Michael Kelly for thoughtful discussions; Jonathan Ashwell, John O'Shea, Nicholas Restifo, Eytan Ruppin, and Xin Wang for critical reading of the manuscript; and David Goldstein, Mariam Malik, and the NCI Office of Science and Technology Resources for their support. This work used the NIH high-performance computing cluster and was supported by the Intramural Research Program of the National Cancer Institute, Center for Cancer Research, NIH.

AUTHOR CONTRIBUTIONS

A.M., J.N., T.C., S.H., and R.B. designed the research. A.M. designed and developed computational (bioinformatic) pipelines. A.M., J.N., and T.C. performed research and analyzed data. S.T. guided TIL isolation procedures. D.B.M. provided advice on LCMV biology and LCMV viral stocks. M.M., Y.Z., and B.T. contributed to scRNA-seq capture. A.M. and R.B. wrote the manuscript with contributions from J.N. and S.H. S.H. and R.B. supervised the research.

DECLARATION OF INTERESTS

The authors declare no competing interests.

OPEN
ACCESS
CellPress

Received: February 19, 2019
Revised: August 21, 2019
Accepted: October 30, 2019
Published: December 3, 2019

REFERENCES

Aarntzen, E.H., De Vries, I.J., Lesterhuis, W.J., Schuurhuis, D., Jacobs, J.F., Bol, K., Schreibelt, G., Mus, R., De Wilt, J.H., Haanen, J.B., et al. (2013). Targeting CD4(+) T-helper cells improves the induction of antitumor responses in dendritic cell-based vaccination. Cancer Res. *73*, 19–29.

Agata, Y., Kawasaki, A., Nishimura, H., Ishida, Y., Tsubata, T., Yagita, H., and Honjo, T. (1996). Expression of the PD-1 antigen on the surface of stimulated mouse T and B lymphocytes. Int. Immunol. *8*, 765–772.

Ahmadzadeh, M., Pasetto, A., Jia, L., Deniger, D.C., Stevanović, S., Robbins, P.F., and Rosenberg, S.A. (2019). Tumor-infiltrating human CD4$^+$ regulatory T cells display a distinct TCR repertoire and exhibit tumor and neoantigen reactivity. Sci. Immunol. *4*, eaao4310.

Ahrends, T., Spanjaard, A., Pilzecker, B., Babala, N., Bovens, A., Xiao, Y., Jacobs, H., and Borst, J. (2017). CD4$^+$ T Cell Help Confers a Cytotoxic T Cell Effector Program Including Coinhibitory Receptor Downregulation and Increased Tissue Invasiveness. Immunity *47*, 848–861.

Alspach, E., Lussier, D.M., and Schreiber, R.D. (2019). Interferon γ and Its Important Roles in Promoting and Inhibiting Spontaneous and Therapeutic Cancer Immunity. Cold Spring Harb. Perspect. Biol. *11*, a028480.

Azizi, E., Carr, A.J., Plitas, G., Cornish, A.E., Konopacki, C., Prabhakaran, S., Nainys, J., Wu, K., Kiseliovas, V., Setty, M., et al. (2018). Single-Cell Map of Diverse Immune Phenotypes in the Breast Tumor Microenvironment. Cell *174*, 1293–1308.

Beatty, G., and Paterson, Y. (2001). IFN-gamma-dependent inhibition of tumor angiogenesis by tumor-infiltrating CD4+ T cells requires tumor responsiveness to IFN-gamma. J. Immunol. *166*, 2276–2282.

Belkaid, Y., Mendez, S., Lira, R., Kadambi, N., Milon, G., and Sacks, D. (2000). A natural model of Leishmania major infection reveals a prolonged “silent” phase of parasite amplification in the skin before the onset of lesion formation and immunity. J. Immunol. *165*, 969–977.

Bluestone, J.A., Mackay, C.R., O’Shea, J.J., and Stockinger, B. (2009). The functional plasticity of T cell subsets. Nat. Rev. Immunol. *9*, 811–816.

Bono, M.R., Fernández, D., Flores-Santibáñez, F., Rosemblatt, M., and Sauma, D. (2015). CD73 and CD39 ectonucleotidases in T cell differentiation: Beyond immunosuppression. FEBS Lett. *589*, 3454–3460.

Borst, J., Ahrends, T., Bąbała, N., Melief, C.J.M., and Kastenmüller, W. (2018). CD4$^+$ T cell help in cancer immunology and immunotherapy. Nat. Rev. Immunol. *18*, 635–647.

Bos, R., and Sherman, L.A. (2010). CD4+ T-cell help in the tumor milieu is required for recruitment and cytolytic function of CD8+ T lymphocytes. Cancer Res. *70*, 8368–8377.

Brummelman, J., Mazza, E.M.C., Alvisi, G., Colombo, F.S., Grilli, A., Mikulak, J., Mavilio, D., Alloisio, M., Ferrari, F., Lopci, E., et al. (2018). High-dimensional single cell analysis identifies stem-like cytotoxic CD8$^+$ T cells infiltrating human tumors. J. Exp. Med. *215*, 2520–2535.

Buja, A., and Eyuboglu, N. (1992). Remarks on Parallel Analysis. Multivariate Behav. Res. *27*, 509–540.

Butler, A., Hoffman, P., Smibert, P., Papalexi, E., and Satija, R. (2018). Integrating single-cell transcriptomic data across different conditions, technologies, and species. Nat. Biotechnol. *36*, 411–420.

Carlson, C.M., Endrizzi, B.T., Wu, J., Ding, X., Weinreich, M.A., Walsh, E.R., Wani, M.A., Lingrel, J.B., Hogquist, K.A., and Jameson, S.C. (2006). Kruppel-like factor 2 regulates thymocyte and T-cell migration. Nature *442*, 299–302.

Carmi, Y., Spitzer, M.H., Linde, I.L., Burt, B.M., Prestwood, T.R., Perlman, N., Davidson, M.G., Kenkel, J.A., Segal, E., Pusapati, G.V., et al. (2015). Allogeneic IgG combined with dendritic cell stimuli induce antitumour T-cell immunity. Nature *521*, 99–104.

Chao, J.L., and Savage, P.A. (2018). Unlocking the Complexities of Tumor-Associated Regulatory T Cells. J. Immunol. *200*, 415–421.

Chihara, N., Madi, A., Kondo, T., Zhang, H., Acharya, N., Singer, M., Nyman, J., Marjanovic, N.D., Kowalczyk, M.S., Wang, C., et al. (2018). Induction and transcriptional regulation of the co-inhibitory gene module in T cells. Nature *558*, 454–459.

Ciucci, T., Vacchio, M.S., Gao, Y., Tomassoni Ardori, F., Candia, J., Mehta, M., Zhao, Y., Tran, B., Pepper, M., Tessarollo, L., et al. (2019). The Emergence and Functional Fitness of Memory CD4$^+$ T Cells Require the Transcription Factor Thpok. Immunity *50*, 91–105.

Corbett, T.H., Griswold, D.P., Jr., Roberts, B.J., Peckham, J.C., and Schabel, F.M., Jr. (1975). Tumor induction relationships in development of transplantable cancers of the colon in mice for chemotherapy assays, with a note on carcinogen structure. Cancer Res. *35*, 2434–2439.

Cousens, L.P., Peterson, R., Hsu, S., Dorner, A., Altman, J.D., Ahmed, R., and Biron, C.A. (1999). Two roads diverged: interferon α/β- and interleukin 12-mediated pathways in promoting T cell interferon γ responses during viral infection. J. Exp. Med. *189*, 1315–1328.

Crawford, A., Angelosanto, J.M., Kao, C., Doering, T.A., Odorizzi, P.M., Barnett, B.E., and Wherry, E.J. (2014). Molecular and transcriptional basis of CD4$^+$ T cell dysfunction during chronic infection. Immunity *40*, 289–302.

Crotty, S. (2015). A brief history of T cell help to B cells. Nat. Rev. Immunol. *15*, 185–189.

Crotty, S. (2019). T Follicular Helper Cell Biology: A Decade of Discovery and Diseases. Immunity *50*, 1132–1148.

De Simone, M., Arrigoni, A., Rossetti, G., Gruarin, P., Ranzani, V., Politano, C., Bonnal, R.J.P., Provasi, E., Sarnicola, M.L., Panzeri, I., et al. (2016). Transcriptional Landscape of Human Tissue Lymphocytes Unveils Uniqueness of Tumor-Infiltrating T Regulatory Cells. Immunity *45*, 1135–1147.

DeNardo, D.G., Barreto, J.B., Andreu, P., Vasquez, L., Tawfik, D., Kolhatkar, N., and Coussens, L.M. (2009). CD4(+) T cells regulate pulmonary metastasis of mammary carcinomas by enhancing protumor properties of macrophages. Cancer Cell *16*, 91–102.

Duhen, T., Duhen, R., Montler, R., Moses, J., Moudgil, T., de Miranda, N.F., Goodall, C.P., Blair, T.C., Fox, B.A., McDermott, J.E., et al. (2018). Co-expression of CD39 and CD103 identifies tumor-reactive CD8 T cells in human solid tumors. Nat. Commun. *9*, 2724.

Gajewski, T.F., Schreiber, H., and Fu, Y.X. (2013). Innate and adaptive immune cells in the tumor microenvironment. Nat. Immunol. *14*, 1014–1022.

Gattinoni, L., Zhong, X.S., Palmer, D.C., Ji, Y., Hinrichs, C.S., Yu, Z., Wrzesinski, C., Boni, A., Cassard, L., Garvin, L.M., et al. (2009). Wnt signaling arrests effector T cell differentiation and generates CD8+ memory stem cells. Nat. Med. *15*, 808–813.

Grover, H.S., Blanchard, N., Gonzalez, F., Chan, S., Robey, E.A., and Shastri, N. (2012). The Toxoplasma gondii peptide AS15 elicits CD4 T cells that can control parasite burden. Infect. Immun. *80*, 3279–3288.

Haber, A.L., Biton, M., Rogel, N., Herbst, R.H., Shekhar, K., Smillie, C., Burgin, G., Delorey, T.M., Howitt, M.R., Katz, Y., et al. (2017). A single-cell survey of the small intestinal epithelium. Nature *551*, 333–339.

Hunder, N.N., Wallen, H., Cao, J., Hendricks, D.W., Reilly, J.Z., Rodmyre, R., Jungbluth, A., Gnjatic, S., Thompson, J.A., and Yee, C. (2008). Treatment of metastatic melanoma with autologous CD4+ T cells against NY-ESO-1. N. Engl. J. Med. *358*, 2698–2703.

Ikushima, H., Negishi, H., and Taniguchi, T. (2013). The IRF family transcription factors at the interface of innate and adaptive immune responses. Cold Spring Harb. Symp. Quant. Biol. *78*, 105–116.

Im, S.J., Hashimoto, M., Gerner, M.Y., Lee, J., Kissick, H.T., Burger, M.C., Shan, Q., Hale, J.S., Lee, J., Nasti, T.H., et al. (2016). Defining CD8+ T cells that provide the proliferative burst after PD-1 therapy. Nature *537*, 417–421.

Iwata, S., Mikami, Y., Sun, H.W., Brooks, S.R., Jankovic, D., Hirahara, K., Onodera, A., Shih, H.Y., Kawabe, T., Jiang, K., et al. (2017). The Transcription

Factor T-bet Limits Amplification of Type I IFN Transcriptome and Circuitry in T Helper 1 Cells. Immunity *46*, 983–991.

Jeannet, G., Boudousquié, C., Gardiol, N., Kang, J., Huelsken, J., and Held, W. (2010). Essential role of the Wnt pathway effector Tcf-1 for the establishment of functional CD8 T cell memory. Proc. Natl. Acad. Sci. USA *107*, 9777–9782.

Joshi, N.S., and Kaech, S.M. (2008). Effector CD8 T cell development: a balancing act between memory cell potential and terminal differentiation. J. Immunol. *180*, 1309–1315.

Kammertoens, T., Friese, C., Arina, A., Idel, C., Briesemeister, D., Rothe, M., Ivanov, A., Szymborska, A., Patone, G., Kunz, S., et al. (2017). Tumour ischaemia by interferon-γ resembles physiological blood vessel regression. Nature *545*, 98–102.

Kowalczyk, M.S., Tirosh, I., Heckl, D., Rao, T.N., Dixit, A., Haas, B.J., Schneider, R.K., Wagers, A.J., Ebert, B.L., and Regev, A. (2015). Single-cell RNA-seq reveals changes in cell cycle and differentiation programs upon aging of hematopoietic stem cells. Genome Res. *25*, 1860–1872.

Kurtulus, S., Madi, A., Escobar, G., Klapholz, M., Nyman, J., Christian, E., Pawlak, M., Dionne, D., Xia, J., Rozenblatt-Rosen, O., et al. (2019). Checkpoint Blockade Immunotherapy Induces Dynamic Changes in PD-1^{-}CD8^{+} Tumor-Infiltrating T Cells. Immunity *50*, 181–194.

Levine, J.H., Simonds, E.F., Bendall, S.C., Davis, K.L., Amir, el-, A.D., Tadmor, M.D., Litvin, O., Fienberg, H.G., Jager, A., Zunder, E.R., et al. (2015). Data-Driven Phenotypic Dissection of AML Reveals Progenitor-like Cells that Correlate with Prognosis. Cell *162*, 184–197.

Liberzon, A., Birger, C., Thorvaldsdóttir, H., Ghandi, M., Mesirov, J.P., and Tamayo, P. (2015). The Molecular Signatures Database (MSigDB) hallmark gene set collection. Cell Syst. *1*, 417–425.

Mackay, L.K., and Kallies, A. (2017). Transcriptional Regulation of Tissue-Resident Lymphocytes. Trends Immunol. *38*, 94–103.

Malandro, N., Budhu, S., Kuhn, N.F., Liu, C., Murphy, J.T., Cortez, C., Zhong, H., Yang, X., Rizzuto, G., Altan-Bonnet, G., et al. (2016). Clonal Abundance of Tumor-Specific CD4(+) T Cells Potentiates Efficacy and Alters Susceptibility to Exhaustion. Immunity *44*, 179–193.

Malchow, S., Leventhal, D.S., Nishi, S., Fischer, B.I., Shen, L., Paner, G.P., Amit, A.S., Kang, C., Geddes, J.E., Allison, J.P., et al. (2013). Aire-dependent thymic development of tumor-associated regulatory T cells. Science *339*, 1219–1224.

Matloubian, M., Concepcion, R.J., and Ahmed, R. (1994). CD4+ T cells are required to sustain CD8+ cytotoxic T-cell responses during chronic viral infection. J. Virol. *68*, 8056–8063.

Mumberg, D., Monach, P.A., Wanderling, S., Philip, M., Toledano, A.Y., Schreiber, R.D., and Schreiber, H. (1999). CD4(+) T cells eliminate MHC class II-negative cancer cells *in vivo* by indirect effects of IFN-gamma. Proc. Natl. Acad. Sci. USA *96*, 8633–8638.

Nish, S.A., Zens, K.D., Kratchmarov, R., Lin, W.W., Adams, W.C., Chen, Y.H., Yen, B., Rothman, N.J., Bhandoola, A., Xue, H.H., et al. (2017). CD4+ T cell effector commitment coupled to self-renewal by asymmetric cell divisions. J. Exp. Med. *214*, 39–47.

Oldstone, M.B. (2002). Biology and pathogenesis of lymphocytic choriomeningitis virus infection. Curr. Top. Microbiol. Immunol. *263*, 83–117.

Ott, P.A., Hu, Z., Keskin, D.B., Shukla, S.A., Sun, J., Bozym, D.J., Zhang, W., Luoma, A., Giobbie-Hurder, A., Peter, L., et al. (2017). An immunogenic personal neoantigen vaccine for patients with melanoma. Nature *547*, 217–221.

Pepper, M., and Jenkins, M.K. (2011). Origins of CD4(+) effector and central memory T cells. Nat. Immunol. *12*, 467–471.

Plitas, G., Konopacki, C., Wu, K., Bos, P.D., Morrow, M., Putintseva, E.V., Chudakov, D.M., and Rudensky, A.Y. (2016). Regulatory T Cells Exhibit Distinct Features in Human Breast Cancer. Immunity *45*, 1122–1134.

Qin, Z., and Blankenstein, T. (2000). CD4+ T cell–mediated tumor rejection involves inhibition of angiogenesis that is dependent on IFN gamma receptor expression by nonhematopoietic cells. Immunity *12*, 677–686.

Ribas, A., and Wolchok, J.D. (2018). Cancer immunotherapy using checkpoint blockade. Science *359*, 1350–1355.

Rosenberg, S.A., and Restifo, N.P. (2015). Adoptive cell transfer as personalized immunotherapy for human cancer. Science *348*, 62–68.

Sade-Feldman, M., Yizhak, K., Bjorgaard, S.L., Ray, J.P., de Boer, C.G., Jenkins, R.W., Lieb, D.J., Chen, J.H., Frederick, D.T., Barzily-Rokni, M., et al. (2018). Defining T Cell States Associated with Response to Checkpoint Immunotherapy in Melanoma. Cell *175*, 998–1013.

Saitoh, T., et al. (2002). Lymphotoxin-L receptor mediates NEMO-independent NF-UB activation. FEBS *532*, 45–51.

Sakaguchi, S., Yamaguchi, T., Nomura, T., and Ono, M. (2008). Regulatory T cells and immune tolerance. Cell *133*, 775–787.

Sena-Esteves, M., et al. (2004). Optimized large-scale production of high titer lentivirus vector pseudotypes. Journal of Virological Methods.

Siddiqui, I., Schaeuble, K., Chennupati, V., Fuertes Marraco, S.A., Calderon-Copete, S., Pais Ferreira, D., Carmona, S.J., Scarpellino, L., Gfeller, D., Pradervand, S., et al. (2019). Intratumoral Tcf1^{+}PD-1^{+}CD8^{+} T Cells with Stem-like Properties Promote Tumor Control in Response to Vaccination and Checkpoint Blockade Immunotherapy. Immunity *50*, 195–211.

Simoni, Y., Becht, E., Fehlings, M., Loh, C.Y., Koo, S.L., Teng, K.W.W., Yeong, J.P.S., Nahar, R., Zhang, T., Kared, H., et al. (2018). Bystander CD8^{+} T cells are abundant and phenotypically distinct in human tumour infiltrates. Nature *557*, 575–579.

Snell, L.M., McGaha, T.L., and Brooks, D.G. (2017). Type I Interferon in Chronic Virus Infection and Cancer. Trends Immunol. *38*, 542–557.

Subramanian, A., Tamayo, P., Mootha, V.K., Mukherjee, S., Ebert, B.L., Gillette, M.A., Paulovich, A., Pomeroy, S.L., Golub, T.R., Lander, E.S., and Mesirov, J.P. (2005). Gene set enrichment analysis: a knowledge-based approach for interpreting genome-wide expression profiles. Proc. Natl. Acad. Sci. USA *102*, 15545–15550.

Sun, H., Lu, B., Li, R.Q., Flavell, R.A., and Taneja, R. (2001). Defective T cell activation and autoimmune disorder in Stra13-deficient mice. Nat. Immunol. *2*, 1040–1047.

Tanaka, A., and Sakaguchi, S. (2017). Regulatory T cells in cancer immunotherapy. Cell Res. *27*, 109–118.

Thommen, D.S., and Schumacher, T.N. (2018). T Cell Dysfunction in Cancer. Cancer Cell *33*, 547–562.

Tian, L., Goldstein, A., Wang, H., Ching Lo, H., Sun Kim, I., Welte, T., Sheng, K., Dobrolecki, L.E., Zhang, X., Putluri, N., et al. (2017). Mutual regulation of tumour vessel normalization and immunostimulatory reprogramming. Nature *544*, 250–254.

Tran, E., Turcotte, S., Gros, A., Robbins, P.F., Lu, Y.C., Dudley, M.E., Wunderlich, J.R., Somerville, R.P., Hogan, K., Hinrichs, C.S., et al. (2014). Cancer immunotherapy based on mutation-specific CD4+ T cells in a patient with epithelial cancer. Science *344*, 641–645.

Trapnell, C., Cacchiarelli, D., Grimsby, J., Pokharel, P., Li, S., Morse, M., Lennon, N.J., Livak, K.J., Mikkelsen, T.S., and Rinn, J.L. (2014). The dynamics and regulators of cell fate decisions are revealed by pseudotemporal ordering of single cells. Nat. Biotechnol. *32*, 381–386.

van der Maaten, L. (2014). Accelerating t-SNE using Tree-Based Algorithms. J. Mach. Learn. Res. *15*, 3221–3245.

van der Maaten, L., and Hinton, G. (2008). Visualizing Data using t-SNE. J. Mach. Learn. Res. *9*, 2579–2605.

Wang, L., Wildt, K.F., Castro, E., Xiong, Y., Feigenbaum, L., Tessarollo, L., and Bosselut, R. (2008). The zinc finger transcription factor Zbtb7b represses CD8-lineage gene expression in peripheral CD4+ T cells. Immunity *29*, 876–887.

Wei, S.C., Levine, J.H., Cogdill, A.P., Zhao, Y., Anang, N.A.S., Andrews, M.C., Sharma, P., Wang, J., Wargo, J.A., Pe'er, D., and Allison, J.P. (2017). Distinct Cellular Mechanisms Underlie Anti-CTLA-4 and Anti-PD-1 Checkpoint Blockade. Cell *170*, 1120–1133.e17.

Wherry, E.J., and Kurachi, M. (2015). Molecular and cellular insights into T cell exhaustion. Nat. Rev. Immunol. *15*, 486–499.

Wu, T., Ji, Y., Moseman, E.A., Xu, H.C., Manglani, M., Kirby, M., Anderson, S.M., Handon, R., Kenyon, E., Elkahloun, A., et al. (2016). The TCF1-Bcl6

axis counteracts type I interferon to repress exhaustion and maintain T cell stemness. Sci. Immunol. *1*, eaai8593.

Yu, F., Sharma, S., Jankovic, D., Gurram, R.K., Su, P., Hu, G., Li, R., Rieder, S., Zhao, K., Sun, B., and Zhu, J. (2018). The transcription factor Bhlhe40 is a switch of inflammatory versus antiinflammatory Th1 cell fate determination. J. Exp. Med. *215*, 1813–1821.

Zhang, L., Yu, X., Zheng, L., Zhang, Y., Li, Y., Fang, Q., Gao, R., Kang, B., Zhang, Q., Huang, J.Y., et al. (2018). Lineage tracking reveals dynamic relationships of T cells in colorectal cancer. Nature *564*, 268–272.

Zheng, C., Zheng, L., Yoo, J.K., Guo, H., Zhang, Y., Guo, X., Kang, B., Hu, R., Huang, J.Y., Zhang, Q., et al. (2017a). Landscape of Infiltrating T Cells in Liver Cancer Revealed by Single-Cell Sequencing. Cell *169*, 1342–1356.

Zheng, G.X., Terry, J.M., Belgrader, P., Ryvkin, P., Bent, Z.W., Wilson, R., Ziraldo, S.B., Wheeler, T.D., McDermott, G.P., Zhu, J., et al. (2017b). Massively parallel digital transcriptional profiling of single cells. Nat. Commun. *8*, 14049.

Zhou, X., Yu, S., Zhao, D.M., Harty, J.T., Badovinac, V.P., and Xue, H.H. (2010). Differentiation and persistence of memory CD8(+) T cells depend on T cell factor 1. Immunity *33*, 229–240.

Zhu, J., Yamane, H., and Paul, W.E. (2010). Differentiation of effector CD4 T cell populations (*). Annu. Rev. Immunol. *28*, 445–489.

STAR★METHODS

KEY RESOURCES TABLE

REAGENT or RESOURCE	SOURCE	IDENTIFIER
Antibodies		
Anti-B220-V500 (Clone RA3-6B2)	BD Pharmigen	Cat# 561227; RRID:AB_10562193
Anti-Cxcr5-PE-eFluor 610 (Clone SPRCL5)	Thermofisher	Cat# 61-7185-82; RRID:AB_2574660
Anti-CD4-BUV737	BD Pharmigen	Cat# 564298, RRID:AB_2732918
Anti-TCRb-BV711	BD Pharmigen	Cat# 563135; RRID:AB_2629564
Anti-Thy1.1-FITC	Thermofisher	Cat# 11-0900-81; RRID:AB_465151
Anti-CD44-AF700	Thermofisher	Cat# 56-0441-82; RRID:AB_494011
Anti-PD-1-PECy7	Thermofisher	Cat# 25-9985-82; RRID:AB_10853805
Anti-CD8b-BUV395	BD Pharmigen	Cat# 563786; RRID:AB_2732919
Anti-CD45.2- BV786	BD Pharmigen	Cat# 563686; RRID:AB_2738375
Siglec F-BV510	BD Pharmigen	Cat# 740158; RRID:AB_2739911
NK1.1-BV510	BD Pharmigen	Cat# 563096; RRID:AB_2738002
CD11b-BV510	BD Pharmigen	Cat# 562950; RRID:AB_2737913
CD11c-BV510	BD Pharmigen	Cat# 562949; RRID:AB_2732056)
IL7R-APC	Thermofisher	Cat# 17-1271-82; RRID:AB_469435
Ccr7-PE	Thermofisher	Cat# 12-1979-42; RRID:AB_10670625
Ccr7-PE-Cy7	Thermofisher	Cat# 25-1971-82; RRID:AB_469652
Bcl6-BV421	BD Pharmigen	Cat# 563363; RRID:AB_2738159
Lag3-APC-ef780	Thermofisher	Cat# 47-2231-82; RRID:AB_2637323
Foxp3-ef450	Thermofisher	Cat# 48-5773-82; RRID:AB_1518812
Foxp3-AF488	Thermofisher	Cat# 53-5773-82; RRID:AB_763537
Granzyme B-PE	Innovative Research	Cat# MHGB04; RRID:AB_10372671
Tbet-PE-Cy7	Thermofisher	Cat# 25-5825-82; RRID:AB_11042699
CD200-APC-R700	BD Pharmigen	Cat# 565546; RRID:AB_2739288
CD39-super bright 600	Thermofisher	Cat# 63-0391-82; RRID:AB_2717037
IRF7-PE	Thermofisher	Cat# 12-5829-82; RRID:AB_2572629
CD278-BV421	BioLegend	Cat# 313523; RRID:AB_2562538
PD-1-PE-Cy7	Thermofisher	Cat# 25-9985-82; RRID:AB_10853805
PD-1-BU395	BD Pharmigen	Cat# 744549; RRID:AB_2742320
TIM3-PE	BioLegend	Cat# 119704; RRID:AB_345378
CD94-FITC	Thermofisher	Cat# 11-0941-82; RRID:AB_465161
NKG2A/NKG2C/NKG2E-Percp-ef710	Thermofisher	Cat# 46-5896-82; RRID:AB_10853352
IFNγ-FITC	BioLegend	Cat# 505806; RRID:AB_315400
Bacterial and Virus Strains		
LCMV Armstrong	McGavern Lab	
LCMV Clone 13	McGavern Lab	
Biological Samples		
MC38 tumor samples	This paper	
MC38-GP tumor samples	This paper	
Chemicals, Peptides, and Recombinant Proteins		
I-A^b LCMV GP66-77 (DIYKGVYQKSV) tetramer-PE	NIH tetramer facility	
H-$2D^b$ LCMV GP33-42 (KAVYNFATM) tetramer- APC	NIH tetramer facility	
PMA	Sigma-Aldrich	Cat# P8139
Ionomycin	Sigma-Aldrich	Cat# I 0634-1mg
DAPI (4',6-Diamidino-2-Phenylindole, Dihydrochloride)	Life Technologies	Cat# D1306

(*Continued on next page*)

Continued

REAGENT or RESOURCE	SOURCE	IDENTIFIER
Protein Transport Inhibitor	BD Pharmigen	Cat# 554724
I-A^b *Toxoplasma gondii* AS15 (KAVYNFATM) tetramer-ef450	NIH tetramer facility	
Critical Commercial Assays		
eBioscience Transcription Staining Buffer Set	Invitrogen	Cat# 00-5523-00
Fixable Viability Dye UV	Invitrogen	Cat# 65-0868-18
Deposited Data		
scRNaseq: single cell expression profiles of CD4$^+$ T cells	This paper	GSE124691
scRNaseq of Human liver cancer TILs	Zheng et al., 2017a	GSE98638
scRNaseq of Human melanoma TILs	Sade-Feldman et al., 2018	GSE120575
Experimental Models: Cell Lines		
MC38-GP murine colon cancer cell lines	This paper	
MC38 murine colon cancer cell lines	Jack Greiner's lab	
Experimental Models: Organisms/Strains		
C57BL/6Ncr (CD45.2)	Charles River	Charles River 556
Oligonucleotides		
LCMV-GP-F: 5′ GGATCC ATGGGTCAGATTG TGACAATGTTTG 3′	This paper	
LCMV-GP-R: 5′ GCGGCCGCTCAGCGTCTTTT CCAGACGGTTTTTAC 3′	This paper	
Recombinant DNA		
pMRX	Saitoh, 2002	
pHCMV-LCMV-Arm53b	Sena-Esteves, 2004	Addgene 15796
Software and Algorithms		
RobustSingleCell	This paper	https://github.com/asmagen/RobustSingleCell
Flowjo 10.0	Flowjo	https://www.flowjo.com
10X Genomics Cellranger toolkit v2.0.1	10X Genomics	http://software.10xgenomics.com/single-cell/overview/welcome
Prism 7	Graphpad	https://www.graphpad.com/scientific-software/prism/
R	R	https://www.r-project.org
Seurat cell alignment v2.0.1	Butler et al., 2018	https://satijalab.org/seurat/immune_alignment.html

LEAD CONTACT AND MATERIALS AVAILABILITY

Further information and requests should be directed to and will be fulfilled by the Lead Contact, Remy Bosselut (remy.bosselut@nih.gov).

All unique/stable reagents generated in this study are available from the Lead Contact with a completed Materials Transfer Agreement

EXPERIMENTAL MODEL AND SUBJECT DETAILS

Mice

6-12 weeks C57BL/6 mice were purchased from Charles River laboratories and housed in specific pathogen-free facilities. ScRNaseq was performed on male mice for sequencing consistency; flow cytometry was performed indifferently on male and female mice, with no observable difference. Animal procedures were approved by the NCI Animal Care and Use Committee.

Cell Lines and Constructs

MC38 murine colon cancer cell lines (Corbett et al., 1975) were obtained from Jack Greiner's lab and cultured in DMEM that contained 10% heat-inactivated FCS, 0.1 mM nonessential amino acids, 1 mM sodium pyruvate, 0.292mg/ml L-glutamine, 100 pg/ml

streptomycin, 100 U/mL penicillin, 10mM HEPES. MC38-GP cells were generated as follows: LCMV-*gp* gene was amplified from pHCMV-LCMV-Arm53b (addgene#15796) and inserted into pMRX-IRES-Thy1.1 by BamH1 and Not1 (Saitoh, 2002; Sena-Esteves, 2004). Then pMRX-Thy1.1 contained LCMV-*gp* gene was transfected into Plat E cell to package retrovirus. MC38 cell line was transduced by above retrovirus collection and followed by single cell sorting in 96-well plate after 48hs. The monoclonal cell lines were identified by flow cytometry and western blot.

METHOD DETAILS

LCMV Infection Model and Tumor Model

2×10^5 pfu of LCMV Armstrong (Matloubian et al., 1994) were injected intra-peritoneal in 6-12 weeks old C57BL/6 mice. Mice were analyzed 7 days post infection. 2×10^6 pfu of LCMV Clone 13 were injected intra-venously in 6-12 weeks old C57BL/6 mice. Mice were analyzed 21 days post infection. MC38 and MC38-GP tumor cells (0.5×10^6) were subcutaneously injected into the flank of C57BL/6 mice.

Cell Preparation and Flow Cytometry

Lymph node and spleen were prepared and stained as previously described (Wang et al., 2008). For TIL preparation, tumors were dissected 14 to 18 days post-injection, washed in HBSS, cut into small pieces, and subjected to enzymatic digestion with 0.25mg/ml liberase (Roche) and 0.5mg/ml DNAase I (SIGMA) for 30 minutes at 37 degrees. The resulting material were passed through 70um filters and pelleted by centrifugation at 1500rpm. Cell pellets were resuspended in 44% Percoll (GE Healthcare) on an underlay of 67% Percoll, and centrifuged for 20min at 1600 rpm without brake. TILs were isolated from the 44%/67% Percoll interface. Following isolation, cells were blocked with anti-FcγRIII/FcγRII (unconjugated, 2.4G2) and subsequently stained for flow cytometry. Staining for AS15:I-A^b tetramer (Grover et al., 2012), GP66:I-A^b tetramer and Cxcr5 was performed at 37 degrees for 1 hour prior to staining for other cell surface markers. For intracellular staining, cell surface staining were preformed first, following fixation using the Foxp3-staining kit (eBioscience). For cytokine staining, cells were incubated in the presence of PMA (25ng/ml)/Ionomycin (1ug/ml) and Golgi stop for 3 hours, followed by surface staining and intracellular staining. Flow cytometry data were acquired on LSR Fortessa cytometers (BD Biosciences) and analyzed with FlowJo (TreeStar) software. Dead cells and doublets were excluded by LiveDead staining (Invitrogen) and forward scatter height by width gating. Purification of lymphocytes by cell sorting was performed on a FACS Aria or FACS Fusion (BD Biosciences).

Single-Cell RNA-Seq

For each of the two separate biological replicates, 3000-13000 T cells sorted from one Arm infected and 10 tumor-bearing mice were loaded on the Chromium platform (10X Genomics) and libraries were constructed with a Single Cell 3′ Reagent Kit V2 according to the manufacturer instruction. Libraries were sequenced on multiple runs of Illumina NextSeq using paired-end 26x98bp or 26x57bp to reach a sequencing saturation greater than 70% resulting in at least 49000 reads/cell. Cell recovery rate averaged 19%.

QUANTIFICATION AND STATISTICAL ANALYSIS

Experimental Data

Flow cytometry was analyzed using FlowJo 10.5.0. GraphPad Prism (Version 7) was used for graphical representation and statistical analysis of cytometry data. Flow cytometry data are presented as mean ± SEM. Unpaired two-sided Student's T–test was used throughout to measure statistical significance of protein expression by flow cytometry. Statistical significance annotation is denoted in figure legends.

scRNA-Seq Data Pre-processing

De-multiplexing, alignment to the mm10 transcriptome and unique molecular identifier (UMI) calculation were performed using the 10X Genomics Cellranger toolkit (v2.0.1, http://software.10xgenomics.com/single-cell/overview/welcome). Pre-processing, dimensionality reduction and clustering analyses procedures were applied to each dataset (that is, specific tissue origin in each experiment) independently to account for dataset-specific technical variation such as sequencing depth and biological variation in population composition, as follows. We filtered out low quality cells with fewer than 500 detected genes (those with at least one mapped read in the cell). Potential doublets were defined as cells with number of detected genes or number of UMIs above the 98th quantile (top 2% owing to up to 2% estimated doublets rate in the 10X Chromium system). Potentially senescent cells (more than 10% of the reads in the cell mapped to 13 mitochondrial genes) were also excluded. Cell numbers pre- and post-filtering are found in Table S1. Library size (LS_j, number of UMIs in cell j) normalization and natural log transformation were applied to each cell library, i.e., $norm_j^i = \ln(10000 \times (raw_j^i / LS_j) + 1)$, to quantify the expression of gene i in cell j, where raw_j^i is the number of reads for gene i in cell j.

Transcriptomic Effects of TCR Engagement as a Result of GP66-Tetramer-Based Purification

GP66-tetramer binding results in potential cross-linking of and signaling by the TCR of GP66-specific T cells. To model the transcriptomic effect of TCR engagement as a result of GP66-tetramer-based purification, we sought to compare Arm-specific $CD4^+$ T cells

obtained either after GP66-tetramer purification or without tetramer-based purification. To enrich in such cells without tetramer staining, we noted that ~94% of GP66-specific CD4$^+$ splenocytes from Arm-infected mice express little or no IL7R [IL-7 receptor α chain] (Figure S2F). Thus, we considered that most CD44^{hi}CD4$^+$IL7R$^+$ splenocytes were not Arm-specific, and sorted CD44^{hi} IL7R$^-$ (Arm IL7R$^-$) T cells for scRNaseq; in addition to antigen-specific CD44^{hi} GP66-tetramer purified (Arm GP66$^+$) T cells (Figure S2G). Pooled clustering of the two samples revealed 2 (out of 6) clusters heavily dominated by tetramer-stained cells (Figure S2H, top), suggesting that the bias introduced by tetramer staining was limited to those clusters. As expected from GP66 tetramer engagement with the TCR, GP66-specific clusters were characterized by genes involved in T cell receptor signaling and NFKB signaling (Table S6), while clusters containing cells from both samples displayed features of Tfh and Th1 cells (Figure S2H, bottom). We designated the GP66-characteristic genes as the TCR engagement GP66 signature (Table S3) and regressed the activation scores of the signature from the expression matrix using a linear regression model fitted to each gene.

Dimensionality Reduction

Highly variable genes were defined as genes with greater than one standard deviation of the dispersion from the average expression of each gene. However, to account for heteroscedasticity, variable genes were identified separately in bins defined based on average expression. PCA analysis was performed on the normalized expression of the set of dataset-specific highly variable genes. We selected the top PCs based on gene permutation test (Buja and Eyuboglu, 1992). 'Barnes-hut' approximate version of t-SNE (van der Maaten, 2014) (perplexity set to 30, 10k iterations) was applied on the top PCs to obtain a 2D projection of the data for visualization.

Gene Signature Activation Quantification

Gene signature activation was quantified relative to a technically similar background gene set as described in (Haber et al., 2017). Briefly, we identify the top 10 most similar (nearest neighbors) genes in terms of average expression and variance, then define the signature activation as the average expression of the signature genes minus the average expression of the background genes. The GP66 tetramer staining signature is defined above. Additionally, we defined lists of ribosomal, mitochondrial, and cell cycle genes (Kowalczyk et al., 2015) for confounder controls (Table S3).

High-Resolution Clustering

Phenograph clustering (Levine et al., 2015) using the top PCs (see dimensionality reduction) was performed independently on each dataset to allow full control of the clustering resolution based on dataset-specific coverage and heterogeneity features. The clustering resolution (number of clusters) is controlled by the K nearest neighbor (KNN) parameter. We designed a simulation analysis to estimate the optimal clustering resolution, i.e., at what resolution the clustering is superior in quality to clustering driven by technical biases inherent to scRNaseq, as follows. Here we define the clustering quality as the clustering modularity reported by Phenograph, which indicates intra-cluster compactness and inter-cluster separation. The simulations consist of repeating the clustering analysis on 100 shuffled expression matrices to estimate the 'null' distribution of the clustering quality, where the gene expression measurements are permuted within each cell to retain the cell-specific coverage biases. We repeated this process for varying value of the KNN parameter k to compare the clustering modularity of the original O_k to the shuffled S_k data. The final resolution was defined as the maximal resolution where $(O_k / S_k) \geq 2$. Following this strategy, k was set to 22, 27, 29, 22, 64 and 51, for dLN experiment 1 and 2, TILs experiment 1 and 2 and Arm experiment 1 and 2, respectively. All clustering analysis was performed for each sample separately, except of the low-resolution clustering of TILs and dLN (Figure S2B), where dLN and TILs from experiment 1 were analyzed jointly via pooled clustering. Pooled clustering analysis (joint rather than separated by dataset) and visualization was performed using PCA on the aggregate list of highly variable genes defined on each dataset. Clustering was done with and without controlling for confounding factors (number of UMIs, number of detected genes and gene signatures activation of ribosomal, mitochondrial, cell cycle and GP66 staining signature). Clustering analysis of TILs, dLN, and Arm cells showed little overlap even after correcting for potential confounders.

After obtaining the initial clusters and identifying the overexpressed genes in each cluster, we apply two filters: (1) we exclude small clusters of B cells (CD79$^+$ populations) from each dataset. (2) We identify PCs driven by B cell marker genes and remove the individual cells whose expression profile has high scores for those PCs (outliers). We then repeat the entire processing and clustering to prevent detecting highly variable genes and PCs driven by contaminations, which may in turn reduce the signal of other small populations of interest.

Differential Expression Analysis and Population Matching

Differential expression was performed using Limma (version 3.32.10). Cluster defining genes reported throughout passed FDR-corrected (Benjamini–Hochberg procedure) differential expression tests independently in the two replicates (Fold change > 1.25, Q-value < 0.1). Statistical significance is indicated in relevant figures and supplementary tables. We initially performed differential expression analysis between each cluster against the pool of all other clusters within a given dataset. Identified clusters were labeled as a known T cell subtype if the majority of the known subtype-defining genes were differentially overexpressed in that cluster. We then matched populations across experiments to assess the reproducibility of the populations and to uncover similarities across datasets that are masked due to overall tissue-context-specific differences. To reduce the effects of tissue-context-specific effects on

the similarity calculation, we used the fold change (FC) measure of each gene $FC_g^c = (\langle foreground_g \rangle / \langle background_g \rangle)$ (average of gene *g* in cluster *c* (foreground) relative to all other clusters (background) of the same dataset). Then we measured the Pearson correlation between the FC vectors of all pairs of clusters across datasets. Unlike common batch correction approaches which match the mean and variance of each gene across batches, the FC vectors describe the deviation intensity of each gene relative to the sample mean, without skewing those deviations to match across samples, thereby not removing biologically relevant factors. We compare this approach with an alternative approach that uses Euclidean distances between the average expression vectors, defined as average expression of all genes in a cluster and a recent data integration approach (Butler et al., 2018) following tutorial specifications [https://satijalab.org/seurat/immune_alignment.html; version 2.0.1].

Robust Cluster Calling and Robust Population Comparisons

For each dataset, we defined 'robust clusters' as those that had highly similar match in the biological replicate. High similarity is defined as Pearson correlation coefficient greater than ~1.28 standard deviations from the mean for each dataset, corresponding to nominal p value of 0.1. Hierarchical clustering was performed on the identified robust clusters using the inter-cluster similarity matrix, where the similarity was defined as above using the Pearson correlation between the FC vectors. Using the vector of average expression vectors did not achieve similar result; specifically, using hierarchical clustering of the Euclidean distances between the clusters average expression vector retained the grouping of clusters based on origin tissue (Figure S3B). We then analyzed differential expression patterns for clusters belonging to each meta-cluster, excluding cell cycle clusters. For a given pair of clusters of interest, A and B in datasets X and Y respectively, we performed three differential expression analyses: (1) differential expression in A relative to other clusters in X, (2) differential expression in B relative to other clusters in Y, and (3) differential expression in A relative to B. In addition to average expression differences, we quantified the detection rate of gene X as proportion of cells where 1 or more reads was mapped to X and prioritized differentially expressed genes exhibiting also differential detection across conditions. This analysis was performed for the two replicates separately and the results interpreted jointly; a gene was deemed as overexpressed in cluster A in tissue X if it is overexpressed relative to other clusters in X as well as relative to B, in both replicates.

scRNaseq Contour Plots

Normalized scRNaseq expression measurements were visualized as contours, where zero (0) values were assigned random value drawn from a normal distribution centered around 0.

scRNaseq Violin Plots

Violin plots were used to visualize the scaled expression distribution per cluster or group of clusters, where scaled expression corresponds to Min-Max scaling of the normalized UMIs to the range of [0,1], i.e., $X_{norm} = (X - X_{min} / X_{max} - X_{min})$, where X_{min} and X_{max} are the minimum and maximum value, respectively, of each gene.

Reversed Graph Embedding

Trajectory analysis of TIL populations (group I and II, excluding group III Tregs) was performed using Monocle (version 2.9.0, parameters max_components = 2, method = DDRTree).

Gene Signature Definition

For each TIL subpopulation (group I Th1, group II Isc, group II nRes and group III Treg) we selected overexpressed genes exhibiting differential detection (as defined above) relative to all other TILs across both experiments (Table S3).

Correspondence to Human Data

Human liver cancer TIL scRNaseq counts were downloaded from GEO [GSE98638]. Non-CD4$^+$ T cells were filtered based on the classification in the original publication (Zheng et al., 2017a). Human gene symbols were translated to Mouse gene symbols using package biomaRt (version 2.37.8). Pre-processing, clustering, and population matching analysis were applied as described above. Human melanoma TILs data scRNaseq counts were downloaded from GEO [GSE120575]. We selected CD4$^+$ T cells as cells with at least one mapped read to CD4 and [CD3D or CD3E or CD3G], following the authors definition (Sade-Feldman et al., 2018). 108 out of 136 Isc signature genes were mapped to human gene symbols. The detection rate of each Isc signature gene (as defined above) in each lesion were used to assess differential detection across responders and non-responders. We used two-sided Wilcoxon test to quantify the significance of differential activation.

Correspondence with External Gene Signatures

Gene set enrichment analysis of immunologic gene signatures was performed using MSigDB (Liberzon et al., 2015) [C7: immunologic signatures database with clusterProfiler package (version 3.4.3). All other gene signatures were downloaded from the original publication's supplementary materials. Correspondence to Tcmp signature was performed by differential expression of dLN *Ccr7*$^+$ clusters n5-6 relative to other dLN and TIL (n1, n7-8, t1-7) rather than dLN subpopulations alone to satisfy the background conditions

used in the original publication. The heterogeneity of the IL-27 co-inhibitory gene signature (Chihara et al., 2018) was evaluated by analyzing differential gene expression across Th1, Isc, and Treg TIL, indicating which genes are preferentially expressed in one sub-population versus the others.

DATA AND CODE AVAILABILITY

The accession number for the sequencing data (scRNAseq) reported in this paper is GEO:GSE124691.

The computational pipeline is available on https://github.com/asmagen/RobustSingleCell. The code is archived by Zenodo and can be cited via https://doi.org/10.5281/zenodo.3239269.

The pipeline requires access to Slurm high-performance computing core for efficient simulation analyses.

Cell Reports, Volume *29*

Supplemental Information

Single-Cell Profiling Defines Transcriptomic Signatures Specific to Tumor-Reactive versus Virus-Responsive $CD4^+$ T Cells

Assaf Magen, Jia Nie, Thomas Ciucci, Samira Tamoutounour, Yongmei Zhao, Monika Mehta, Bao Tran, Dorian B. McGavern, Sridhar Hannenhalli, and Rémy Bosselut

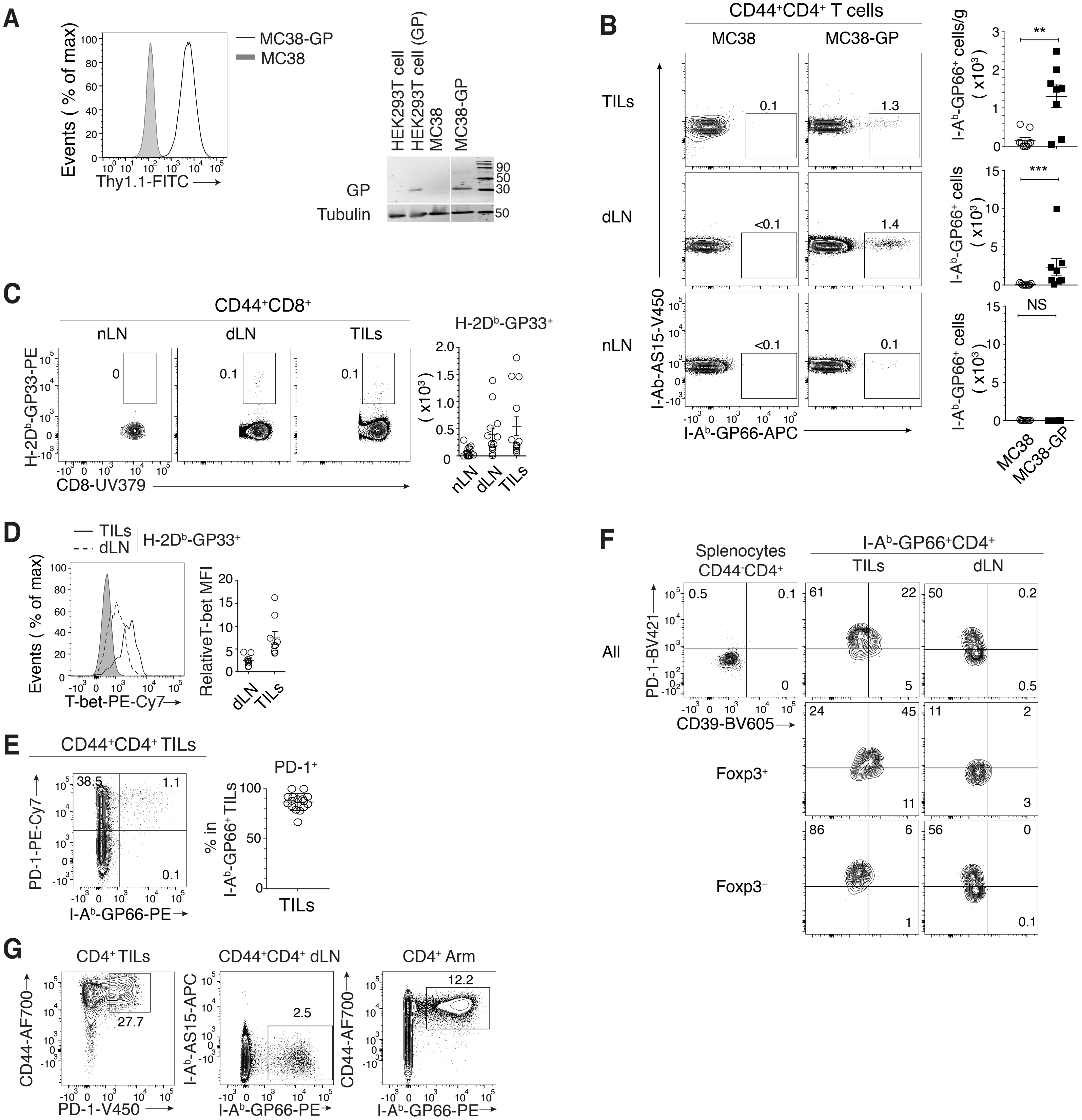

A
MC38-GP
MC38
Events (% of max)
Thy1.1-FITC
HEK293T cell
HEK293T cell (GP)
MC38
MC38-GP
GP
Tubulin
90
50
30
50
B
CD44+CD4+ T cells
MC38
MC38-GP
TILs
dLN
nLN
0.1
1.3
<0.1
1.4
<0.1
0.1
I-Ab-AS15-V450
I-Ab-GP66-APC
I-Ab-GP66+ cells/g (x10³)
I-Ab-GP66+ cells (x10³)
**
***
NS
MC38
MC38-GP
C
CD44+CD8+
nLN
dLN
TILs
H-2Db-GP33-PE
CD8-UV379
0
0.1
0.1
H-2Db-GP33+
(x10³)
D
TILs
dLN
H-2Db-GP33+
Events (% of max)
T-bet-PE-Cy7
Relative T-bet MFI
E
CD44+CD4+ TILs
PD-1-PE-Cy7
I-Ab-GP66-PE
38.5
1.1
0.1
PD-1+
% in I-Ab-GP66+ TILs
TILs
F
Splenocytes CD44-CD4+
I-Ab-GP66+CD4+
TILs
dLN
All
Foxp3+
Foxp3–
PD-1-BV421
CD39-BV605
0.5
0.1
0
61
22
5
50
0.2
0.5
24
45
11
11
2
3
86
6
1
56
0
0.1
G
CD4+ TILs
CD44+CD4+ dLN
CD4+ Arm
CD44-AF700
PD-1-V450
27.7
I-Ab-AS15-APC
I-Ab-GP66-PE
2.5
12.2

Figure S1. Characterization of antigen-specific $CD4^+$ T cell responses in MC38 colon adenocarcinoma tumors, Related to Figure 1.

(**A**) Overlaid protein expression of Thy1.1 in MC38 and MC38-GP cells (left). Immunoblot analysis of GP protein expression in HEK293T cells, HEK293T cells transfected with pMRX-GP-IRES-Thy1.1 plasmid, MC38 cells or MC38-GP cells (right).

(B) C57BL/6 mice were subcutaneously injected MC38 or MC38-GP cells and analyzed at day 14 post-injection. Left panel shows flow cytometry contour plots of GP66 vs. control (AS15 peptide from *T. gondii*) class II tetramer staining in TILs, dLN and nLN from MC38 and MC38-GP tumor-bearing mice. Right panel shows the number of $GP66^+$ TILs per gram of tumor and total number of $GP66^+$ dLN and nLN cells, separately for MC38 and MC38-GP tumor-bearing mice (Unpaired Mann-Whitney U test, ** $p < 0.01$, *** $p < 0.001$, NS: not significant). Data is from 8 mice of each condition analyzed in two experiments.

(C) Flow cytometry analysis of CD8 expression vs. GP33 class I tetramer staining in nLN ,dLN and TILs from MC38-GP tumor-bearing mice (left). Total number of $GP33^+$ dLN and nLN cells, and number of $GP33^+$ TILs per gram of tumor (right). Data is from 13 mice analyzed in three experiments.

(D) Overlaid protein expression of Tbet in $GP33^+$ $CD8^+$ dLN and TILs; grey shaded histogram shows T-bet expression in naive $CD8^+$ splenocytes from tumor-free control mice; histogram shows concatenated data from 6 individual mice analyzed in one experiment. Graph on the right shows T-bet MFI in $GP33^+$ dLN and TILs; data is from 9 tumor bearing mice analyzed in two experiment and is expressed relative to naive $CD8^+$ splenocytes from tumor-free control mice. Each symbol represents an individual mouse.

(E) Flow cytometry contour plots of GP66 tetramer vs. PD-1 staining in TILs (left) and percentage of $PD\text{-}1^+$ cells out of $GP66^+$ TILs (right). Data is from 16 mice analyzed in three experiments.

(F) Right two columns show flow cytometry expression of PD-1 vs. CD39 in $GP66^+CD4^+$ TILs or dLN, gated on all such cells (top row) or $Foxp3^+$or $Foxp3^-$ subsets (bottom rows). Top left plot shows staining of naïve splenocytes from tumor-free mice. Data is representative from 11 mice analyzed in three experiments.

(G) GP66-specific $CD44^{hi}$ $CD4^+$ splenocytes were isolated from WT animals 7 days post-infection with LCMV Armstrong. Protein expression contour of populations used for scRNAseq captures from MC38-GP tumor-bearing mice (TILs PD-1 vs. CD44, dLN GP66 vs. AS15 control) and LCMV Armstrong infected mice (GP66 vs. CD44). Data is representative from >50 mice analyzed in >10 experiments.

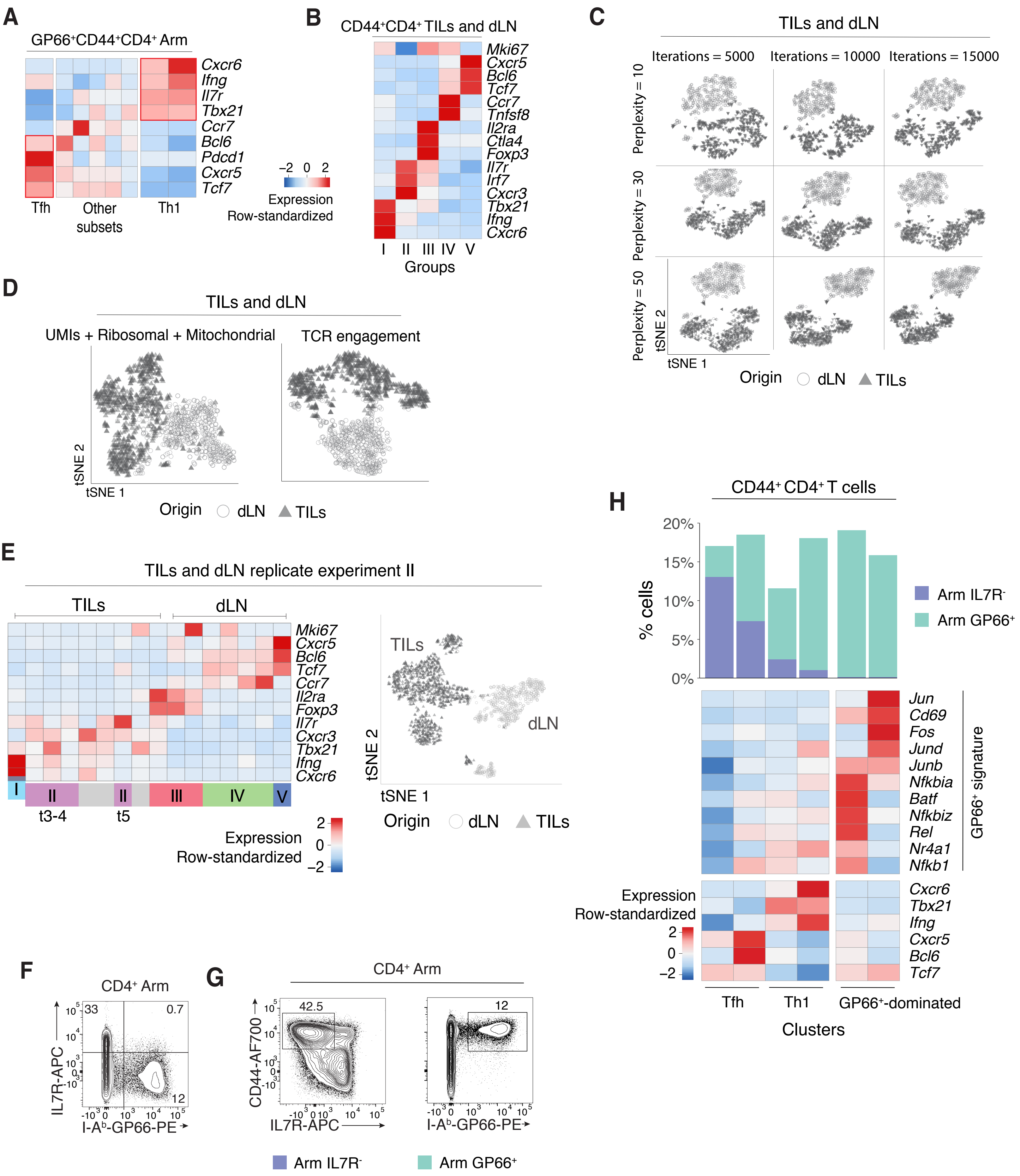

A
GP66+CD44+CD4+ Arm
Cxcr6
Ifng
Il7r
Tbx21
Ccr7
Bcl6
Pdcd1
Cxcr5
Tcf7
Tfh
Other subsets
Th1
−2 0 2
Expression
Row-standardized
B
CD44+CD4+ TILs and dLN
Mki67
Cxcr5
Bcl6
Tcf7
Ccr7
Tnfsf8
Il2ra
Ctla4
Foxp3
Il7r
Irf7
Cxcr3
Tbx21
Ifng
Cxcr6
I II III IV V
Groups
C
TILs and dLN
Iterations = 5000
Iterations = 10000
Iterations = 15000
Perplexity = 10
Perplexity = 30
Perplexity = 50
tSNE 2
tSNE 1
Origin dLN TILs
D
TILs and dLN
UMIs + Ribosomal + Mitochondrial
TCR engagement
tSNE 2
tSNE 1
Origin dLN TILs
E
TILs and dLN replicate experiment II
TILs
dLN
Mki67
Cxcr5
Bcl6
Tcf7
Ccr7
Il2ra
Foxp3
Il7r
Cxcr3
Tbx21
Ifng
Cxcr6
I II II III IV V
t3-4
t5
Expression
Row-standardized
2 0 −2
TILs
dLN
tSNE 2
tSNE 1
Origin dLN TILs
F
CD4+ Arm
33
0.7
12
IL7R-APC
I-Ab-GP66-PE
G
CD4+ Arm
42.5
12
CD44-AF700
IL7R-APC
I-Ab-GP66-PE
Arm IL7R-
Arm GP66+
H
CD44+ CD4+ T cells
20%
15%
10%
5%
0%
% cells
Arm IL7R-
Arm GP66+
Jun
Cd69
Fos
Jund
Junb
Nfkbia
Batf
Nfkbiz
Rel
Nr4a1
Nfkb1
GP66+ signature
Cxcr6
Tbx21
Ifng
Cxcr5
Bcl6
Tcf7
Expression
Row-standardized
2 0 −2
Tfh
Th1
GP66+-dominated
Clusters

Figure S2. Characterization of immune responses to Arm and MC38-GP by scRNAseq, Related to figures 1 and 2.

(A) GP66-specific $CD4^+$ splenocytes from WT animals 7 days post-infection with LCMV Armstrong analyzed by scRNAseq. Heatmap shows row-standardized expression of selected genes across Arm clusters.

(B-E) TILs and dLN cells from WT mice at day 14 post MC38-GP injection analyzed by scRNAseq. **(B)** Heatmap shows row-standardized expression of selected genes across main TIL and dLN groups (as defined in text). **(C)** tSNE display of TILs and dLN cells generated using different parameter combination of perplexity and number of iterations, grey-shaded by tissue origin. **(D)** tSNE displays of TILs and dLN cells, grey-shaded by tissue origin, post confounder correction for number of unique molecular identifiers (UMIs) and expression of ribosomal and mitochondrial coding genes (left) or TCR engagement on dLN cells as a result of GP66-tetramer-based purification (right). **(E)** scRNAseq analysis of TILs and dLN cells from replicate experiment II. Heatmap shows row-standardized expression of selected genes across TIL and dLN clusters (left). tSNE display of TILs and dLN cells, grey-shaded by tissue origin (right).

(F-H) Analysis of $CD4^+$ splenocytes from C57BL/6 animals 7 days post-infection with LCMV Armstrong (Arm). **(F)** Flow cytometry contour plot of GP66 tetramer staining vs. IL7R in $CD4^+$ Arm cells. **(G)** Flow cytometry contour plots of IL7R vs. CD44 (for Arm $IL7R^-$ sample, **left**) and GP66 vs. CD44 (for Arm $GP66^+$ sample, **right**). **(H)** Arm $IL7R^-$ and Arm $GP66^+$ cells analyzed by scRNAseq. Heatmap shows row-standardized expression of selected genes across pooled Arm $IL7R^-$ and Arm $GP66^+$ clusters (**bottom**). Bar plot indicates the number of Arm $IL7R^-$ and Arm $GP66^+$ cells in each cluster relative to the total number of cells (**top**).

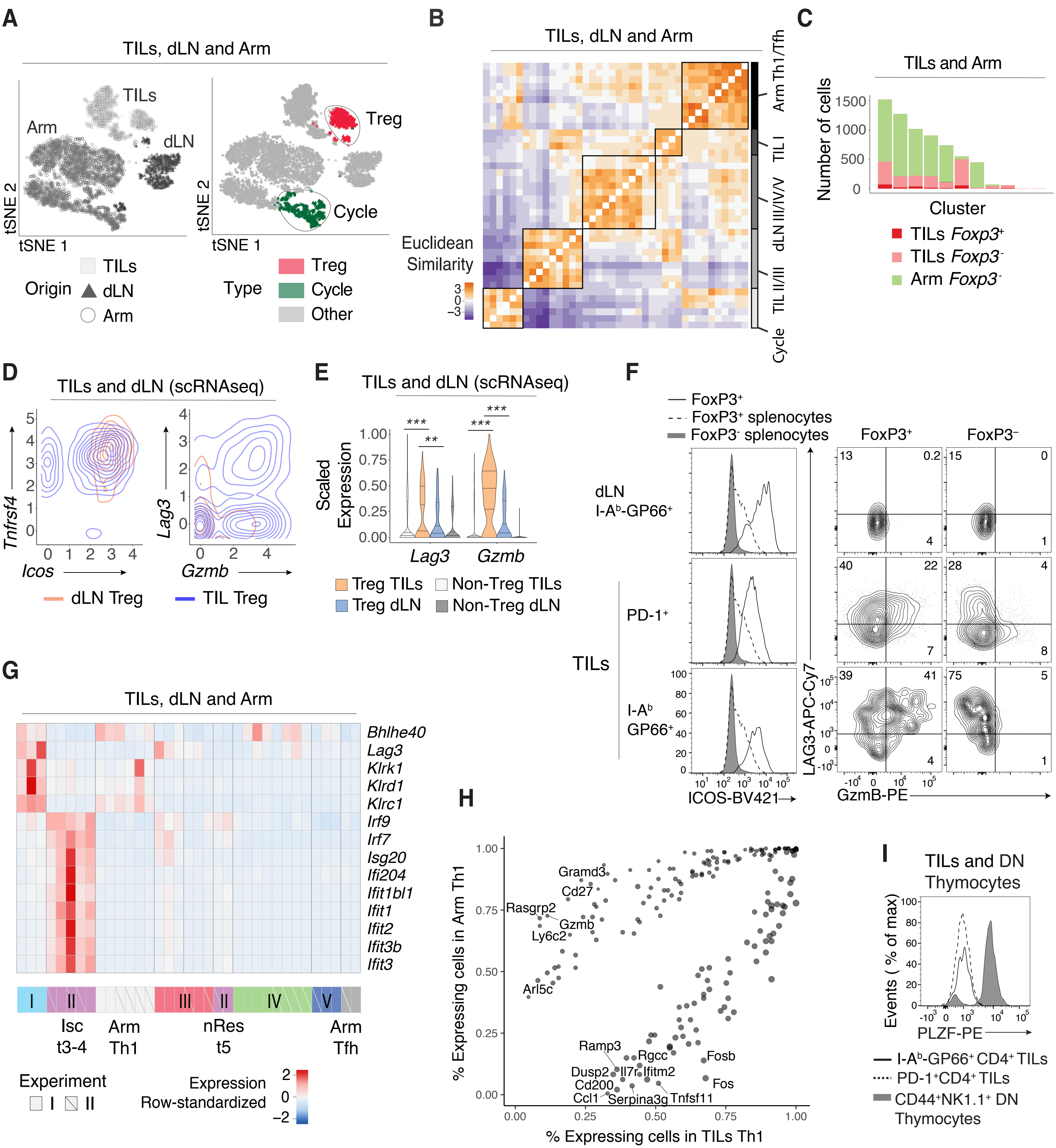

A
TILs, dLN and Arm
TILs
Arm
dLN
tSNE 2
tSNE 1
Origin
TILs
dLN
Arm
Treg
Cycle
Type
Treg
Cycle
Other
B
TILs, dLN and Arm
Euclidean Similarity
Arm Th1/Tfh
TIL I
dLN III/IV/V
TIL II/III
Cycle
C
TILs and Arm
Number of cells
Cluster
TILs Foxp3+
TILs Foxp3-
Arm Foxp3-
D
TILs and dLN (scRNAseq)
Tnfrsf4
Icos
Lag3
Gzmb
dLN Treg
TIL Treg
E
TILs and dLN (scRNAseq)
Scaled Expression
Lag3
Gzmb
Treg TILs
Non-Treg TILs
Treg dLN
Non-Treg dLN
F
FoxP3+
FoxP3+ splenocytes
FoxP3- splenocytes
FoxP3+
FoxP3–
dLN
I-Ab-GP66+
PD-1+
TILs
I-Ab GP66+
ICOS-BV421
LAG3-APC-Cy7
GzmB-PE
G
TILs, dLN and Arm
Bhlhe40
Lag3
Klrk1
Klrd1
Klrc1
Irf9
Irf7
Isg20
Ifi204
Ifit1bl1
Ifit1
Ifit2
Ifit3b
Ifit3
Isc t3-4
Arm Th1
nRes t5
Arm Tfh
Experiment
Expression Row-standardized
H
% Expressing cells in Arm Th1
% Expressing cells in TILs Th1
Gramd3
Cd27
Rasgrp2
Gzmb
Ly6c2
Arl5c
Ramp3
Rgcc
Fosb
Dusp2
Il7r
Ifitm2
Cd200
Fos
Ccl1
Serpina3g
Tnfsf11
I
TILs and DN Thymocytes
Events (% of max)
PLZF-PE
I-Ab-GP66+ CD4+ TILs
PD-1+CD4+ TILs
CD44+NK1.1+ DN Thymocytes

Figure S3. Assessment of tissue-context-specific effects on clustering analyses and TILs-dLN heterogeneity, Related to Figure 3.

(A-C, G,H) TILs, dLN and Arm cells from replicate experiments I and II analyzed by scRNAseq. **(A)** tSNE plots show TILs, dLN, and Arm cells, grey-shaded by origin (left) or color-coded by Treg or cell-cycle (Cycle) clustering assignment (grey for all other clusters) (right). **(B)** Heatmap shows Euclidean similarity between cluster-specific average expression vectors (as defined in text) (left) annotated with cluster origin and cluster group or type (right). **(C)** Bar plot shows relative cluster composition of Foxp3^{+} or Foxp3^{-} TILs and Foxp3^{-} Arm (no Foxp3^{+} cells found in GP66^{+} Arm) after applying a data integration approach (Butler et al., 2018).

(D-E) Comparison of dLN Tregs and TIL Tregs (respectively clusters t6-7 and n1 as shown in Fig. 1A). **(D)** Contour plots of dLN Treg (orange) or TIL Treg (blue) cell distribution according to scRNAseq-detected normalized expression of *Icos* vs. *Tnfrsf4* (left) and *Gzmb* vs. *Lag3* (right). **(E)** Violin plot of *Lag3* and *Gzmb* scRNAseq expression in Treg vs. non-Treg TIL and dLN populations (Unpaired T test, ** $p < 0.01$, *** $p < 0.001$); bands indicate quartiles (25th, 50th and 75th quantile).

(F) Overlaid flow cytometry expression of ICOS in Foxp3^{+} TILs and dLN cells and Foxp3^{+} or Foxp3^{-} CD4^{+} splenocytes from tumor-free control mice (left). Data is representative from 11 mice analyzed in three experiments. Flow cytometry contour plots of Granzyme B vs. LAG3 in Foxp3^{+} TILs or Foxp3^{+} dLN cells (middle), and Foxp3^{-} TILs or Foxp3^{-} dLN (right). Graphs were generated from 5 concatenated files, each of them representing a single mouse and processed in parallel in one experiment. Data is representative of 2 such experiments.

(G) Heatmap shows row-standardized expression of TIL Isc and Th1 characteristic genes across TIL, dLN and Arm clusters.

(H) Gene detection statistics (% expressing cells) of differentially expressed genes by scRNAseq in TILs Th1 and Arm Th1.

(I) Overlaid protein expression of PLZF in GP66^{+} and PD-1^{+} TILs and CD44^{hi} NK1.1^{+} DN (double negative CD4^{-}CD8^{-}) thymocytes from tumor-free control mice. Data is representative from 10 mice analyzed in two experiments.

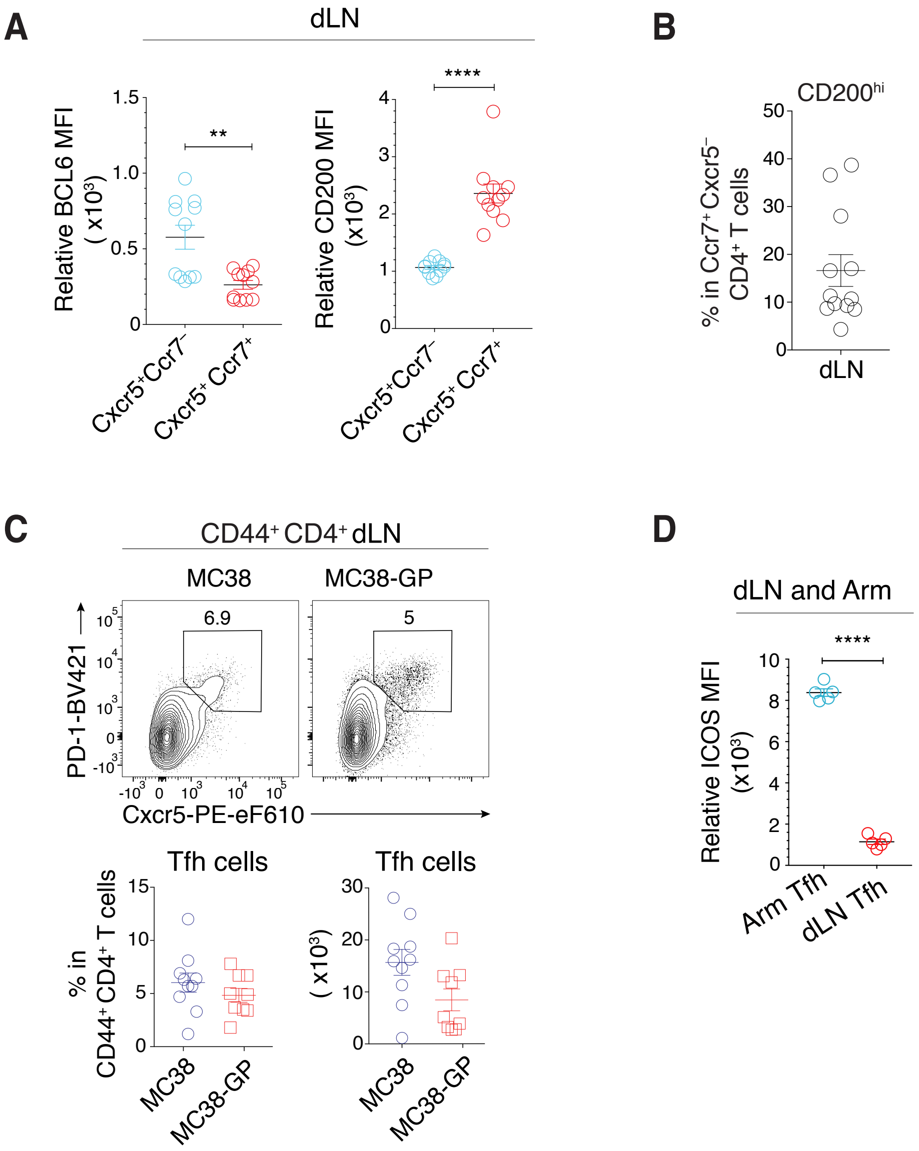

A
dLN
Relative BCL6 MFI (x10³)
1.5
1.0
0.5
0
**
Cxcr5⁺Ccr7⁻
Cxcr5⁺Ccr7⁺
Relative CD200 MFI (x10³)
4
3
2
1
0
****
Cxcr5⁺Ccr7⁻
Cxcr5⁺Ccr7⁺
B
CD200hi
% in Ccr7⁺Cxcr5⁻ CD4⁺ T cells
50
40
30
20
10
0
dLN
C
CD44⁺CD4⁺dLN
MC38
MC38-GP
6.9
5
PD-1-BV421
Cxcr5-PE-eF610
Tfh cells
% in CD44⁺CD4⁺ T cells
15
10
5
0
MC38
MC38-GP
Tfh cells
(x10³)
30
20
10
0
MC38
MC38-GP
D
dLN and Arm
Relative ICOS MFI (x10³)
10
8
6
4
2
0
****
Arm Tfh
dLN Tfh

Figure S4. dLN cell diversity, Related to Figure 4.

(A) Mean fluorescence intensity (MFI) of BCL6 and CD200 in $CXCR5^+$ or $CCR7^+GP66^+$ dLN cells relative to naive $CD4^+$ splenocytes from tumor-free control mice. (Unpaired t-test, ** $p < 0.005$, **** $p < 0.0001$).

(B) Percentage of $CD200^{hi}$ cells out of $CCR7^+CXCR5^+$ dLN cells.

(C) Top panel shows flow cytometry contour plots of CXCR5 vs. PD-1 in $CD44^{hi}$ $CD4^+$ dLN cells from MC38 and MC38-GP tumor-bearing mice. Bottom panel shows percentage of Tfh cells out of total $CD44^{hi}$ $CD4^+$ T cells in dLN (left) and total number of Tfh cells (right). (A-C) Data is representative of 17 mice analyzed in three experiments.

(D) Mean fluorescence intensity (MFI) levels of ICOS in Arm Tfh and dLN Tfh relative to naive $CD4^+$ splenocytes from tumor-free control mice (Unpaired t-test, $p < 10^{-5}$). (A-C) Data is representative of 10 mice analyzed in two experiments.

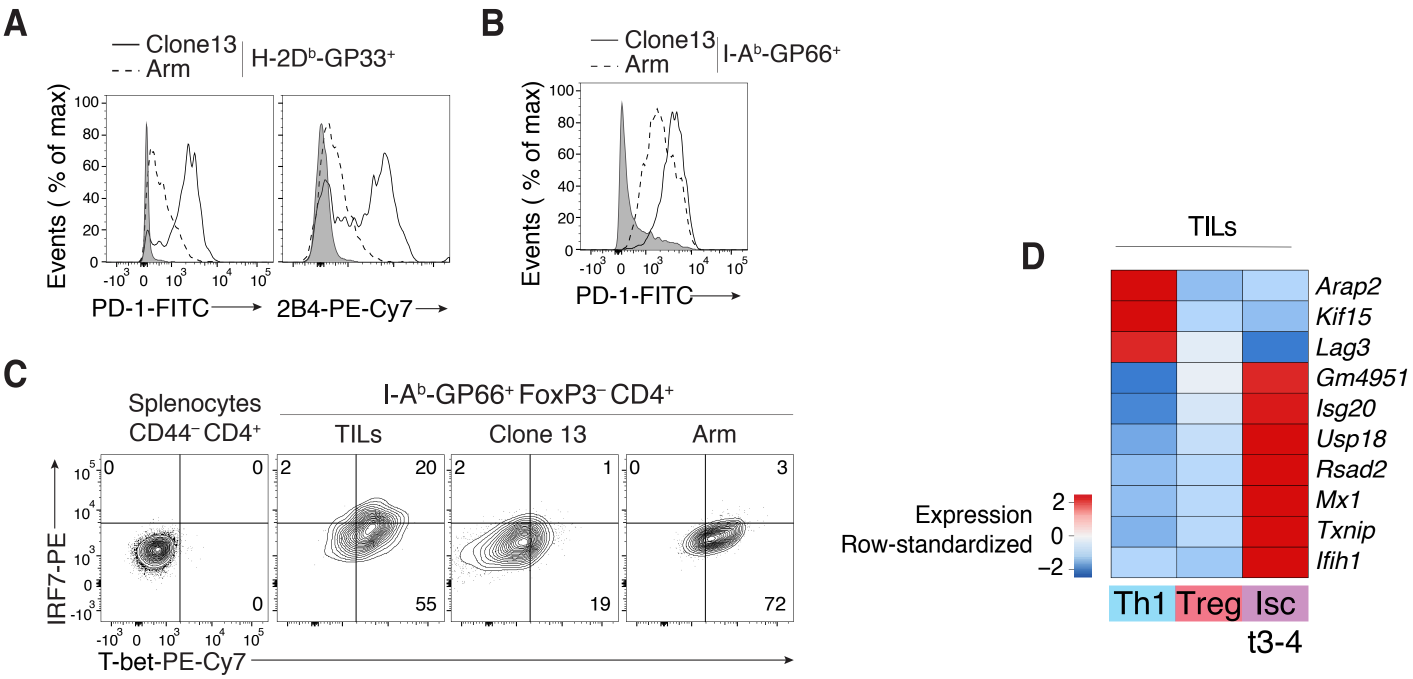
A
Clone13
Arm
H-2D^b-GP33+
Events (% of max)
PD-1-FITC
2B4-PE-Cy7
B
Clone13
Arm
I-A^b-GP66+
Events (% of max)
PD-1-FITC
C
Splenocytes CD44− CD4+
I-A^b-GP66+ FoxP3− CD4+
TILs
Clone 13
Arm
IRF7-PE
T-bet-PE-Cy7
D
TILs
Arap2
Kif15
Lag3
Gm4951
Isg20
Usp18
Rsad2
Mx1
Txnip
Ifih1
Expression
Row-standardized
Th1
Treg
Isc
t3-4

Figure S5. Characterization of the exhaustion status in clone 13 cells and TILs, Related to Figure 5.

(A) Overlaid protein expression of PD-1 or 2B4 in $GP33^+$ $CD8^+$ splenocytes from Arm- or Clone 13-infected mice.

(B) Overlaid protein expression of PD-1 in $GP66^+$ $CD4^+$ splenocytes from Arm- or Clone 13-infected mice. Grey shaded trace (A, B) is control from tumor-free mice.

(C) Flow cytometry contour plots of Tbet vs. IRF7 in Foxp3 $GP66^+$ TILs, Clone 13, or Arm cells, and in naive $CD4^+$ splenocytes from tumor-free control mice. (A-C) Data is representative of 10 mice analyzed in two experiments.

(D) Heatmap shows row-standardized expression of selected exhaustion genes across TIL Th1, Treg and Isc clusters (respectively clusters t1-2, t6-7 and t3-4 as shown in Fig. 1A).

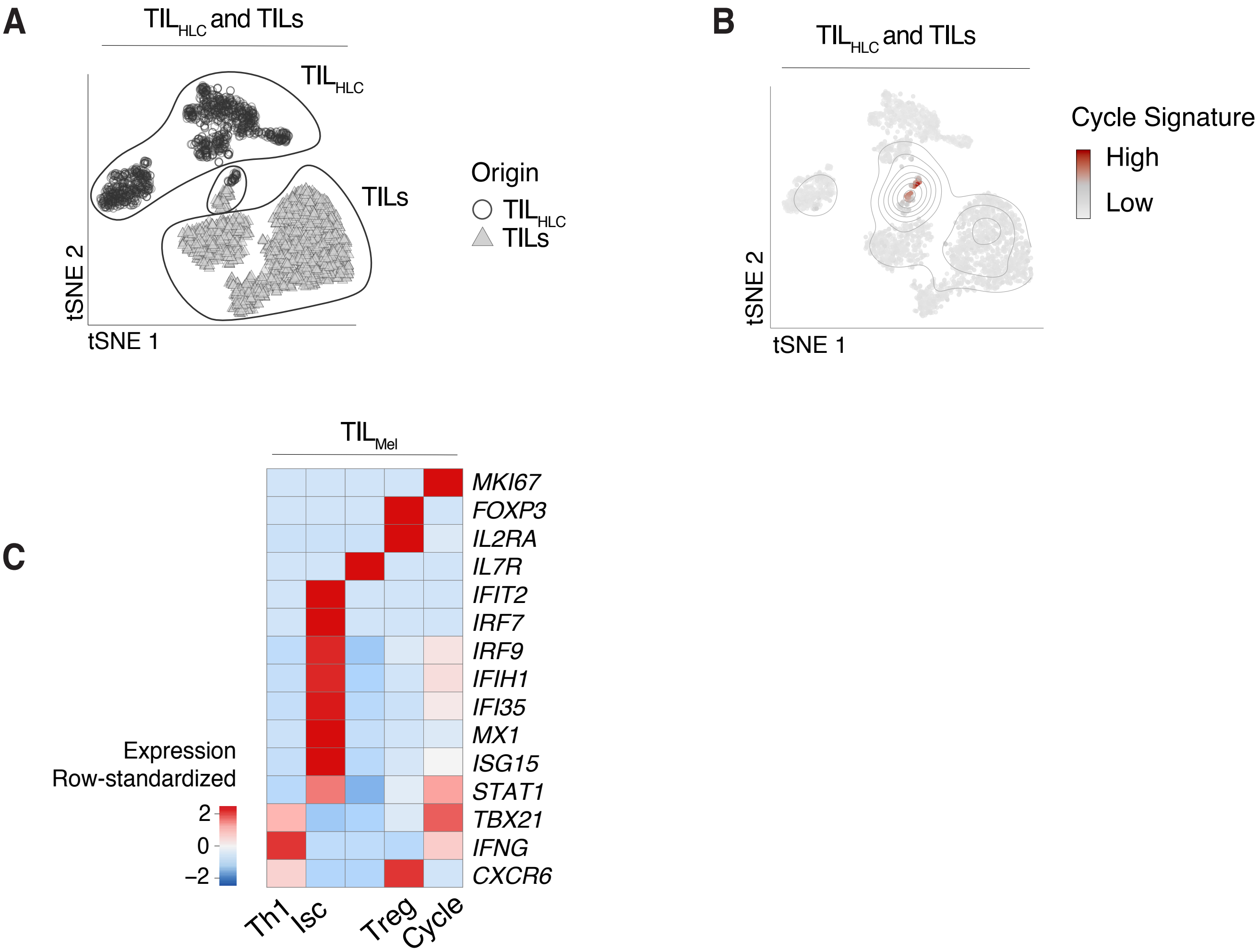

A
TIL HLC and TILs
TIL HLC
TILs
Origin
TIL HLC
TILs
tSNE 2
tSNE 1
B
TIL HLC and TILs
Cycle Signature
High
Low
tSNE 2
tSNE 1
C
TIL Mel
MKI67
FOXP3
IL2RA
IL7R
IFIT2
IRF7
IRF9
IFIH1
IFI35
MX1
ISG15
STAT1
TBX21
IFNG
CXCR6
Expression
Row-standardized
2
0
−2
Th1
Isc
Treg
Cycle

Figure S6. Correspondence to human data and dysfunction gene signatures, Related to Figure 6.

(A-B) Analysis of TIL_{HLC} and TILs (as defined in text). **(A)** tSNE plots show cells grey-shaded by origin. **(B)** tSNE plots show cells color-coded by cell cycle signature activation level.

(C) Analysis of TIL_{Mel} (as defined in text). Heatmap shows row-standardized expression of selected TIL characteristic genes across TIL_{Mel} clusters.
